# Ginsenoside Rg3 Restores Mitochondrial Cardiolipin Homeostasis via GRB2 to Prevent Parkinson's Disease

**DOI:** 10.1002/advs.202403058

**Published:** 2024-08-19

**Authors:** Li‐Feng‐Rong Qi, Shuai Liu, Qiuyuan Fang, Cheng Qian, Chao Peng, Yuci Liu, Peng Yang, Ping Wu, Ling Shan, Qinghua Cui, Qian Hua, Sen Yang, Cunqi Ye, Wei Yang, Ping Li, Xiaojun Xu

**Affiliations:** ^1^ State Key Laboratory of Natural Medicines China Pharmaceutical University Nanjing Jiangsu 210009 China; ^2^ Department of Pharmacy The Fourth Affiliated Hospital Center for Innovative Traditional Chinese Medicine Target and New Drug Research International Institutes of Medicine Zhejiang University School of Medicine Yiwu Zhejiang 322000 China; ^3^ Department of Biophysics and Department of Neurosurgery of the First Affiliated Hospital Zhejiang University School of Medicine Hangzhou Zhejiang 310058 China; ^4^ National Facility for Protein Science in Shanghai Zhangjiang Lab Shanghai Advanced Research Institute Chinese Academy of Science Shanghai 201210 China; ^5^ Shanghai Science Research Center Chinese Academy of Sciences Shanghai 201204 China; ^6^ Dept. Neuropsychiatric Disorders Netherlands Institute for Neuroscience An Institute of the Royal Netherlands Academy of Arts and Sciences Meibergdreef 47 Amsterdam 1105BA the Netherlands; ^7^ Department of Biomedical Informatics School of Basic Medical Sciences Key Laboratory of Molecular Cardiovascular Sciences of the Ministry of Education Center for Non‐Coding RNA Medicine Peking University Health Science Center Beijing Beijing 100191 China; ^8^ School of Life Sciences Beijing University of Chinese Medicine Beijing 100029 China; ^9^ Life Sciences Institute Zhejiang University Hangzhou 310058 China

**Keywords:** cardiolipin, CRLS1, EVI1, GRB2, TRKA

## Abstract

Regulating cardiolipin to maintain mitochondrial homeostasis is a promising strategy for addressing Parkinson's disease (PD). Through a comprehensive screening and validation process involving multiple models, ginsenoside Rg3 (Rg3) as a compound capable of enhancing cardiolipin levels is identified. This augmentation in cardiolipin levels fosters mitochondrial homeostasis by bolstering mitochondrial unfolded protein response, promoting mitophagy, and enhancing mitochondrial oxidative phosphorylation. Consequently, this cascade enhances the survival of tyrosine hydroxylase positive (TH^+^) dopaminergic neurons, leading to an amelioration in motor performance within PD mouse models. Using limited proteolysis–small‐molecule mapping combined with molecular docking analysis, it has confirmed Growth Factor Receptor‐Bound Protein 2 (GRB2) as a molecular target for Rg3. Furthermore, these investigations reveal that Rg3 facilitates the interaction between GRB2 and TRKA (Neurotrophic Tyrosine Kinase, Receptor, Type 1), thus promotes EVI1 (Ecotropic Virus Integration Site 1 Protein Homolog) phosphorylation by ERK, subsequently increases CRLS1 (Cardiolipin Synthase 1) gene expression and boosts cardiolipin synthesis. The absence of GRB2 or CRLS1 significantly attenuates the beneficial effects of Rg3 on PD symptoms. Finally, Tenofovir Disoproxil Fumarate (TDF) that also promotes the binding between GRB2 and TRKA is further identified. The identified compounds, Rg3 and TDF, exhibit promising potential for the prevention of PD by bolstering cardiolipin expression and reinstating mitochondrial homeostasis.

## Graphical abstract

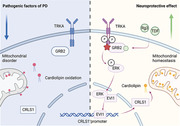

## 1 Introduction

Parkinson's disease (PD) is a progressive neurodegenerative disorder characterized pathologically by the loss of dopaminergic neurons in the substantia nigra (SN) and the presence of protein inclusions known as Lewy bodies.^[^ ^1^ ^]^ Investigations involving both human PD patients and various animal models have revealed the involvement of mitochondrial dysfunction in the pathogenesis of PD.^[^ ^2^ ^]^ Throughout the progression of PD, alpha‐synuclein (αSyn) undergoes misfolding and aggregation on a significant scale, forming amyloid oligomers, ribbons, and fibers that induce neuronal toxicity by disrupting mitochondrial homeostasis.^[^ ^3^ ^]^ Loss‐of‐function mutations in the Parkin RBR E3 Ubiquitin Protein Ligase Protein, PINK1 (PTEN Induced Kinase 1) and Protein DJ‐1 genes impair PINK1/parkin/DJ‐1‐mediated mitophagy, representing another prevalent pathogenic mechanism in PD.^[^ ^4^ ^]^ Furthermore, environmental factors such as 1‐methyl‐4‐phenyl‐1,2,3,6‐tetrahydropyridine (MPTP) and rotenone inhibit the respiratory chain, resulting in diminished ATP production, elevated reactive oxygen species (ROS), and dissipation of the mitochondrial membrane potential. These factors contribute to the exacerbation of mitochondrial dysfunction, culminating in the manifestation of typical PD symptoms.^[^ ^5^ ^]^

Cardiolipin (CL), a pivotal phospholipid in mitochondrial dynamics and function, plays a critical role in shaping and sustaining protein–protein and protein–membrane interactions, while also acting to stabilize the mitochondrial respiratory chain.^[^ ^6^ ^]^ Numerous neurological disorders have been associated with CL abnormalities.^[^ ^7^ ^]^ In a murine model of Alzheimer's disease (AD), a discernible reduction in total CL has been noted, concomitant with mitochondrial synaptic dysfunction.^[^ ^8^ ^]^ Moreover, irregularities in CL content have been associated with compromised mitophagy and a deficiency in complex I activity across various models of PD.^[^ ^9^ ^]^ Multiple investigations highlight an age‐dependent reduction in CL content within murine brains, corresponding to abnormalities in mitochondrial bioenergetics and the depletion of motor neurons.^[^ ^10^ ^]^ CL, a phospholipid predominantly located in the inner mitochondrial membrane, plays a pivotal role in determining mitochondrial fate.^[^ ^11^ ^]^ During mitochondrial stress, CL externalizes onto the outer mitochondrial membrane, exerting a modulatory influence on αSyn to facilitate mitophagy.^[^ ^3^, ^12^ ^]^ Additionally, tau protein exhibits a preference for binding to CL‐enriched domains of the outer mitochondrial membrane, triggering mitochondrial swelling and a decrease in membrane potential.^[^ ^13^ ^]^ Hence, we speculate that CL involved in αSyn, and tau‐induced damage predominantly originates from the externalized pool on the mitochondrial surface, where it undergoes peroxidation and subsequent degradation, leading to a depletion of CL content.^[^ ^11^, ^14^ ^]^ Given the established correlation between CL, mitochondria, and neurological disorders, there is an urgent need to comprehensively elucidate the factors governing CL content. Additionally, investigating whether anomalies in CL content directly initiate pathological processes or manifest as downstream effects is of paramount importance. Small molecules capable of preserving mitochondrial CL content and maintaining its normal distribution hold promise in preventing and mitigating mitochondrial damages. A notable example is elamipretide, which localizes at the inner mitochondrial membrane and interacts with 12 CL‐binding proteins.^[^ ^15^ ^]^ Despite its believed role in stabilizing existing CL, elamipretide proves ineffective in promoting the restoration of CL levels when they are depleted.^[^ ^16^ ^]^ Thus, compounds that increase CL content would be of great interest in this field. Meanwhile, employing these CL increasing compounds as molecular probes, combining chemical biological approaches,^[^ ^17^ ^]^ it is possible to elucidate key targets and novel mechanisms of maintaining CL homeostasis.

Ginsenoside Rg3 (Rg3), a prominent bioactive compound derived from ginseng, demonstrates notable antitumor efficacy across diverse malignancies and exerts neuroprotective effects characterized by its antioxidative and anti‐inflammatory attributes.^[^ ^18^ ^]^ In preclinical models, Rg3 has exhibited enhancements in motor function among rotenone‐induced PD mice.^[^ ^19^ ^]^ The neuroprotective mechanisms of Rg3 partially involve its antioxidative properties, as evidenced by the modulation of glutathione cysteine ligase modulatory subunit and glutathione cysteine ligase regulatory subunit expression, alongside the inhibition of mitochondrial permeability transition pore opening.^[^ ^19^, ^20^ ^]^ However, the specific protein targets through which Rg3 mediates its effects remain to be elucidated.

An essential metazoan adapter protein, Growth Factor Receptor‐Bound Protein 2 (GRB2), plays a crucial role in the receptor tyrosine kinase‐induced activation of RAS/mitogen‐activated protein kinase.^[^ ^21^ ^]^ Specifically, GRB2 directly binds to tropomyosin‐related kinase A (TRKA or neurotrophic receptor tyrosine kinase [NTRK1]).^[^ ^22^ ^]^ In the current study, we observed that the enhanced binding between GRB2 and TRKA induced by Rg3 or Tenofovir Disoproxil Fumarate (TDF) promotes the synthesis of CL. This effect is attributed to the increased expression of Cardiolipin Synthase 1 (CRLS1). This discovery holds significant therapeutic potential for PD, providing a novel and targeted strategy for intervention.

## 2 Results

### 2.1 CL Depletion and Mitochondrial Dysfunction in PD Models

**Figure 1:**
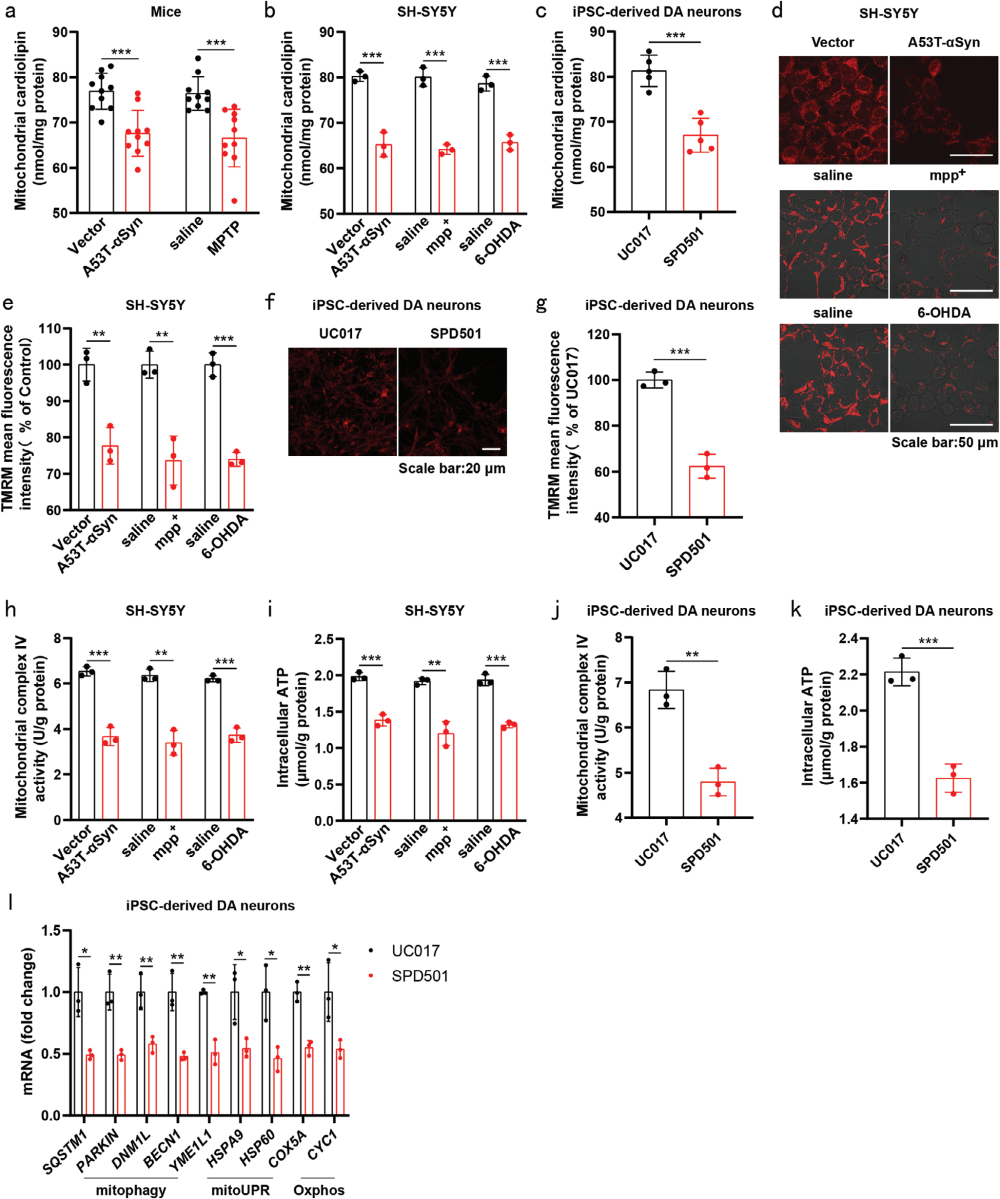
Mitochondrial CL depletion and mitochondrial dysfunction in PD models. a) Mitochondrial CL levels in SN of AAV5‐*Vector*‐ and AAV5‐*A53T‐αSyn*‐injected mice, and in SN of saline‐ and MPTP‐treated mice (20 mg kg^−1^, i.p.) (n = 10 mice per group). SH‐SY5Y cells transfected with pCMV3‐Vector or pCMV3‐A53T‐αSyn‐His for 48 h (b, d, e, h and i). SH‐SY5Y cells were treated with mpp+ (600 µM) or 6‐OHDA (60 µM) for 24 h (b, d, e, h and i). b) Mitochondrial CL levels in SH‐SY5Y cells. Three independent experiments per condition were performed. c) Mitochondrial CL levels in UC017 and SPD501 DA neurons. Five independent experiments per condition were performed. d,e) Mitochondrial membrane potential assessed via TMRM staining in SH‐SY5Y cells. Quantification of mean fluorescence intensity of TMRM is shown. Three independent experiments per condition were performed (scale bar = 50 µm). f,g) Mitochondrial membrane potential assessed via TMRM staining in UC017 and SPD501 DA neurons. Quantification of mean fluorescence intensity of TMRM is shown. Three independent experiments per condition were performed (scale bar = 20 µm). The representative images were obtained from three independent experiments (d and f). h,i) Measurement of mitochondrial cytochrome c oxidase activity (h) and quantification of intracellular ATP concentration (i) in SH‐SY5Y cells. Three independent experiments per condition were performed. j,k) Measurement of mitochondrial cytochrome c oxidase activity (j) and quantification of intracellular ATP concentration (k) in UC017 and SPD501 DA neurons. Three independent experiments per condition were performed. l) mRNA expression of genes related to mitophagy, mitoUPR and Oxphos in UC017 and SPD501 DA neurons. Three independent experiments per condition. Data are normalized to untreated group (e) or UC017 group (g,l). Mean ± standard error of the mean is presented. *p < 0.05, **p < 0.01, ***p < 0.001. Student's two‐tailed unpaired t‐test (a–c, e, g–l). Source data are provided in the Source Data file.

CL levels were evaluated in both in vitro and in vivo models of PD to discern their impact. The overexpression of A53T‐αSyn in SN results in degeneration of the nigrostriatal system, leading to Lewy‐like pathology and motor impairment.^[^ ^23^ ^]^ Additionally, exposure to MPTP causes damage to nigrostriatal dopaminergic neurons.^[^ ^24^ ^]^ In comparison to control mice, the SN of mice treated with either A53T‐αSyn or MPTP exhibited significantly reduced levels of mitochondrial CL (**Figure** **1**a; Figure S1a–c, Supporting Information). Based on current literature, it is conceivable that sustained injury conditions, such as the presence of A53T‐αSyn polymers, could instigate an overabundant externalization of CL from mitochondria, potentially exacerbating mitochondrial membrane permeability and ultimately leading to a reduction in CL levels.^[^ ^3^, ^12^, ^13^ ^]^ Furthermore, mitochondrial CL levels were markedly lower in a group transfected with pCMV3‐*A53T‐αSyn*‐His (Figure 1b). Excess production of ROS and increased oxidative stress can be generated by 1‐methyl‐4‐phenylpyridinium (mpp^+^) and catecholaminergic neurotoxin 6‐hydroxydopamine (6‐OHDA). For in vitro studies on PD, the human neuroblastoma cell line SH‐SY5Y has been widely used, and treatment with mpp^+^ or 6‐OHDA was employed to simulate the disease conditions.^[^ ^25^ ^]^ Mitochondrial CL levels were observed to be lower in the groups treated with mpp^+^ or 6‐OHDA compared to the control group (Figure 1b). To examine the role of CL in PD using human cells, dopaminergic (DA) neurons were differentiated from induced pluripotent stem cells (iPSCs) derived from a healthy donor (UC017) and a patient with sporadic PD (SPD501).^[^ ^26^ ^]^ The successful differentiation of DA neurons was confirmed through the presence of tyrosine hydroxylase (TH), nuclear receptor‐related factor 1 (NURR1), and forkhead box transcription factor A2 (FOXA2), while morphological indicators such as microtubule‐associated protein 2 (MAP‐2), neuronal nuclear protein (NEUN), and TUBULIN were assessed (Figure S1d–f, Supporting Information).^[^ ^26^, ^27^ ^]^ Notably, mitochondrial CL levels were found to be lower in SPD501 DA neurons compared to UC017 DA neurons (Figure 1c). In conclusion, these findings suggest that CL levels are reduced in individuals with PD.

In assessing mitochondrial function in PD, we utilized tetramethylrhodamine (TMRM) staining to assess membrane potential, alongside measurement of mitochondrial cytochrome c oxidase activity and quantification of intracellular ATP concentration to evaluate mitochondrial oxidative phosphorylation (OxPhos) in cellular PD models. Our results revealed a noteworthy reduction in mitochondrial membrane potential in SH‐SY5Y cells transfected with A53T‐αSyn or treated with mpp+ or 6‐OHDA (Figure 1d,e). Furthermore, SPD501 DA neurons demonstrated a diminished mitochondrial membrane potential compared to UC017 DA neurons (Figure 1f,g). SH‐SY5Y cells transfected with A53T‐αSyn or exposed to mpp+ or 6‐OHDA, as well as SPD501 DA neurons, consistently exhibited reduced mitochondrial cytochrome c oxidase activity and diminished intracellular ATP levels (Figure 1h–k). Additionally, the expression of genes associated with mitochondrial unfolded protein response (mitoUPR), mitophagy and mitochondrial Oxphos was notably lower in SPD501 DA neurons when compared to UC017 DA neurons (Figure 1l). These findings collectively imply that the interplay of reduced mitochondrial CL levels and mitochondrial dysfunction holds a pivotal role in the pathogenesis of PD.

### 2.2 Upregulation of CL for Neuroprotection via GRB2

We developed a screening strategy aimed at identifying compounds that restore CL levels for the prevention of PD. In our initial screening, we utilized the 10‐nonyl acridine orange (NAO) fluorescence dye^[^ ^28^ ^]^ to scrutinize a library comprising over 400 natural products^[^ ^29^ ^]^ for compounds that selectively elevate CL levels. Three compounds, namely Phillyrin (Number‐8), Rg3 (Number‐81), and Astragalin (Number‐219), were identified as eliciting increased NAO signals in the *C. elegans* N2 strain (Figure S2a andb, Supporting Information). Contemporary studies suggest that Phillyrin, Rg3, and Astragalin exhibit notable anti‐inflammatory, anti‐cancer, and antioxidative properties.^[^ ^30^ ^]^ In the *C. elegans* BZ555 strain (*dat‐1*p::GFP), Green fluorescent protein (GFP) is selectively expressed in DA neurons under the control of dopamine transporter gene (*dat‐1*) core promoter.^[^ ^31^ ^]^ Following 6‐OHDA treatment, GFP‐positive DA neurons undergo gradual degeneration, resulting in a decline in GFP signals.^[^ ^31^ ^]^ Employing this assay, we found that all the three compounds, which increased CL content, demonstrated a protective effect on DA neurons (Figure S2c and d, Supporting Information). To assess the efficacy of these three compounds in clearing αSyn aggregates, we utilized the transgenic *C. elegans* NL5901 strain (*unc‐54*p::αSynuclein::YFP). The expression of αSyn was measured based on yellow fluorescent protein (YFP) signals.^[^ ^32^ ^]^ Of the three compounds investigated, treatment with Phillyrin and Rg3 notably attenuated the accumulation of αSyn in the NL5901 strain, with Rg3 exhibiting the most pronounced effect (Figure S2e and f, Supporting Information). MitoUPR levels were quantified through GFP signal measurements in the transgenic *C. elegans* SJ4100 strain (*hsp‐6*p::GFP),^[^ ^33^ ^]^ facilitating direct observation. Following exposure to 6‐OHDA, a decline in mitoUPR levels was evident in SJ4100 strain, while administration of Rg3 facilitated the restoration of mitoUPR levels (Figure S2g and h, Supporting Information). In comparison with the solvent control, Rg3 extended the lifespan of the *C. elegans* NL5901 strain (mean = 17.5 days versus 20 days, respectively) (Figure S2i and j, Supporting Information). These results suggest that Rg3 is a potent anti‐PD compound that elevates CL levels and maintains mitochondrial homeostasis.

The preventive efficacy of Rg3 was substantiated in mammalian neural cells. Treatment with Rg3 exhibited a dose‐dependent increase in the viability of SH‐SY5Y cells exposed to mpp^+^, A53T‐αSyn, or 6‐OHDA, as well as SPD501 DA neurons exposed to mpp^+^ (Figure S3a–f, Supporting Information). Additionally, Rg3 facilitated the clearance of overexpressed A53T‐αSyn in SH‐SY5Y cells (Figure S3g and h, Supporting Information). In alignment with in vivo findings in *C. elegans*, a direct quantification of mitochondrial CL revealed that Rg3 treatment reinstated the diminished mitochondrial CL levels in A53T‐αSyn‐overexpression SH‐SY5Y cells or SPD501 DA neurons (Figure S3i and j, Supporting Information). Annexin V labelled with fluorescein isothiocyanate (FITC) can efficiently bind to CL on the surface of the intact and isolated mitochondria.^[^ ^14a^ ^]^ When contrasted with UC017 DA neurons, mitochondria derived from SPD501 DA neurons exhibited an approximately twofold rise in Annexin V‐FITC fluorescence, signifying heightened mitochondrial CL externalization (Figure S3k and l, Supporting Information). Notably, in both UC017 and SPD501 DA neurons, no substantial alteration in mitochondrial Annexin V‐FITC fluorescence was observed following Rg3 treatment, indicating a lack of effect by Rg3 on CL externalization (Figure S3k and l, Supporting Information). Furthermore, Rg3 restored the mitochondrial membrane potential, mitochondrial cytochrome c oxidase activity and intracellular ATP levels in the above‐mentioned neurons (Figure S4a–h, Supporting Information). The expression of genes related to mitoUPR, mitophagy and Oxphos was lower in A53T‐αSyn‐overexpressing SH‐SY5Y cells or SPD501 DA neurons, but was restored upon treatment with Rg3 (Figure S4i and j, Supporting Information). In the murine model of PD, Rg3 administration prevented the A53T‐αSyn‐induced loss of nigral DA neurons (Figure S4k and l, Supporting Information). In neurodegenerative diseases, the documented neuroprotective influence of Rg3 has been reported. Notably, Rg3 has demonstrated the capability to ameliorate oxidative damage induced by rotenone in murine PD model,^[^ ^19^ ^]^ enhance mitochondrial function, and mitigate cognitive impairment in rats exhibiting AD symptoms.^[^ ^34^ ^]^ Our investigation marked a pioneering revelation, elucidating that Rg3 imparts neuroprotective effects through the augmentation of mitochondrial CL levels and the maintenance of mitochondrial homeostasis. Furthermore, the precise mechanism through which Rg3 governs CL was still unclear.

**Figure 2:**
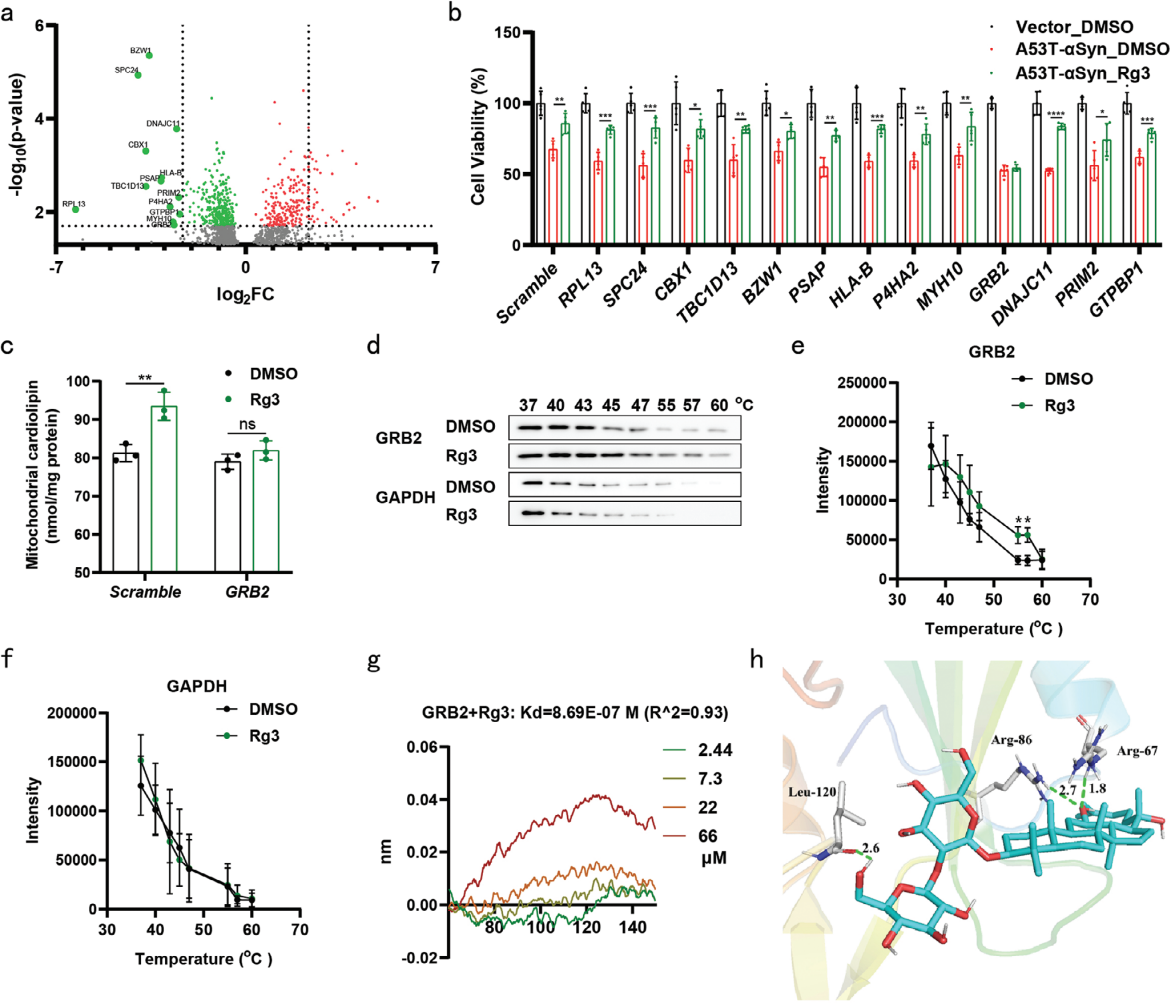
Upregulation of CL for neuroprotection via GRB2. a) A volcano plot illustrates proteins quantified by label‐free mass spectrometry with the potential to bind Rg3. Only proteins identified in two or three replicates, demonstrating with a fold change >5.5 and a *p*‐value < 0.02, were considered as Rg3‐binding proteins. b) SH‐SY5Y cells were treated with Rg3 (5 µM) or DMSO (0.1%) for 24 h after transfection with indicated siRNAs and pCMV3‐*Vector* or pCMV3‐*A53T‐αSyn*‐His for 24 h. Cytotoxicity was assessed using CCK‐8 assay, with five independent experiments per condition. c) Following transfection with Scramble or GRB2 siRNAs for 24 h, SH‐SY5Y cells were treated with Rg3 (5 µM) or DMSO (0.1%) for 24 h. Mitochondrial CL levels were quantified, with three independent experiments per condition. d–f) Soluble protein fraction was heated for denaturation in SH‐SY5Y cells treated with Rg3 (5 µM) or DMSO (0.1%) for 24 h. Representative images of western blotting for GRB2 and GAPDH in soluble protein fraction are shown d). Quantification of GRB2 levels (e) and GAPDH levels (f). g) The binding affinity between Rg3 and GRB2 was determined on a ForteBio Octet system. h) Potential cross‐linking sites for GRB2. The dashed lines represent the distance between amino acid residues and Rg3. Leu‐120, Arg‐67 and Arg‐86 were identified as sites cross‐linked with Rg3 through hydrogen bonds. Data are normalized to Vector_DMSO group (b). Mean ± standard error of the mean is presented. *p < 0.05, **p < 0.01, ***p < 0.001, ****p < 0.0001, ns, not significant. One‐way ANOVA with Tukey's multiple comparisons test (b), two‐way ANOVA with Sidak's multiple comparisons test (c, e, f). Source data are provided in the Source Data file.

Limited proteolysis–small‐molecule mapping (LiP‐SMap) was employed to identify the direct target of Rg3 within proteomes^[^ ^35^ ^]^ (Figure S5a, Supporting Information). A comprehensive identification of 1448 proteins (*p* < 0.05) was achieved through LiP‐SMap (Table S1, Supporting Information). Employing screening criteria of fold change values >5.5 and *p*‐values <0.02, we identified 13 candidate proteins with diminished detection, including RPL13, SPC24, CBX1, TBC1D13, BZW1, PSAP, HLA‐B, P4HA2, MYH10, GRB2, DNAJC11, PRIM2 and GTPBP1 (**Figure** **2**a).

To determine the proteins pivotal for mediating the protective effects of Rg3, SH‐SY5Y cells were transfected with siRNAs targeting the 13 candidate genes (Figure S5b, Tables S2 and S3, Supporting Information). Subsequently, the cells were transfected with A53T‐αSyn‐overexpression plasmids and treated with Rg3. The protective effects of Rg3 against A53T‐αSyn‐induced neural damage were completely abolished only upon silencing of GRB2 (Figure 2b). Direct quantification of mitochondrial CL illustrated that Rg3 treatment restored the diminished levels of mitochondrial CL induced by A53T‐αSyn, an effect that disappeared when GRB2 was silenced (Figure 2c). To determine whether Rg3 directly binds to GRB2, we used the cellular thermal shift assay (CETSA) to perform label‐free target validation; CETSA is based on the principle that ligand binding enhances the thermal stability of target proteins.^[^ ^36^ ^]^ In contrast to GAPDH, Rg3 enhanced the thermal stability of GRB2 in a temperature‐dependent manner (Figure 2d–f). Furthermore, bio‐layer interferometry revealed a dissociation constant *K_D_* 0.869 µM between GRB2 and Rg3 (Figure 2g). Molecular docking of Rg3 onto GRB2 (PDB: 1CJ1) was executed using the Autodock software,^[^ ^37^ ^]^ revealing a singular potential binding pocket surrounding the Arg‐67, Arg‐86 and Leu‐120 residues of the GRB2 SH2 domain (Figure 2h).

### 2.3 Rg3 Enhanced the Interaction between GRB2 and TRKA in Human Neural Cells

**Figure 3:**
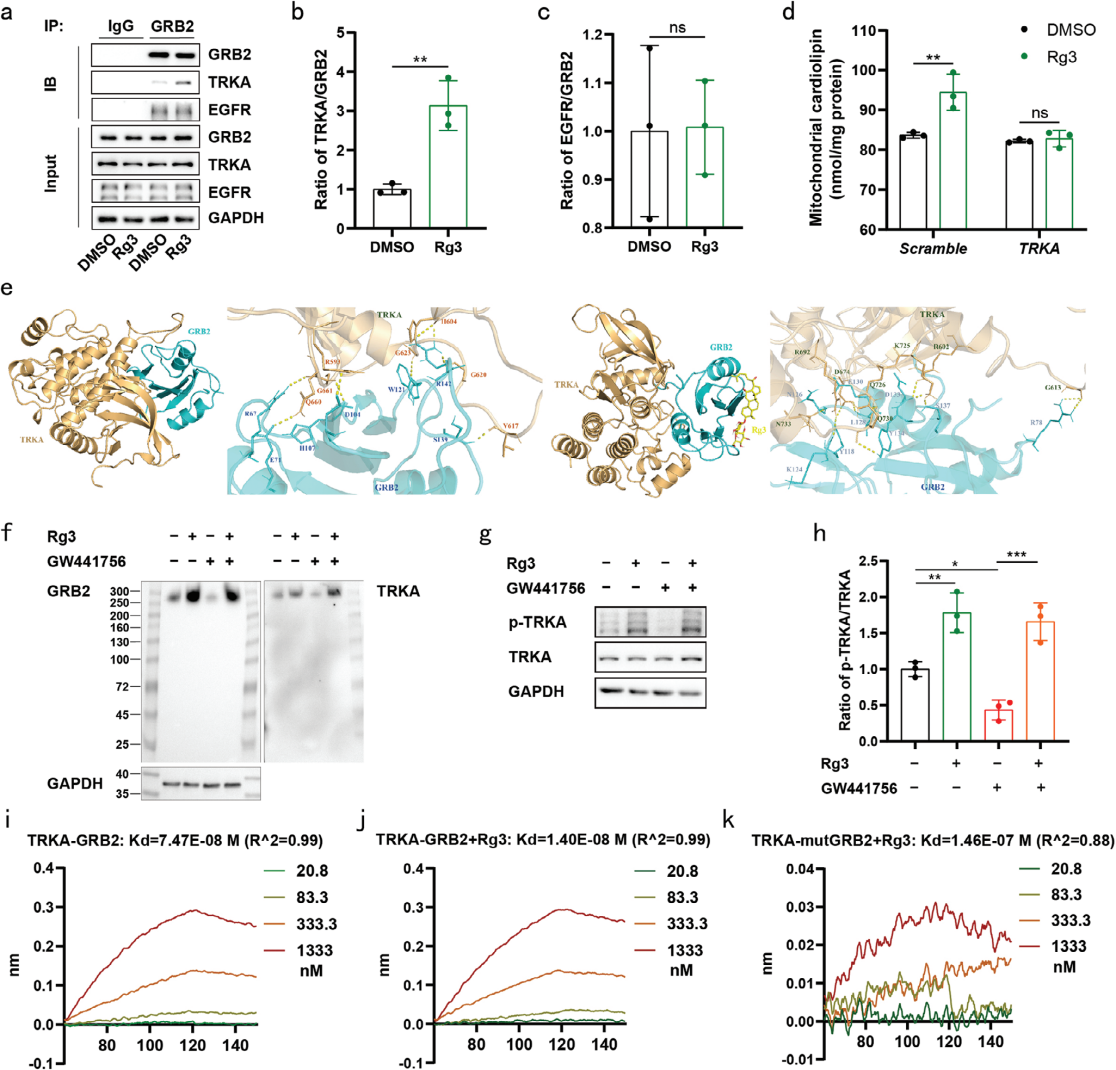
Rg3 enhanced the interaction between GRB2 and TRKA in human neural cells. a–c) SH‐SY5Y cells were treated with Rg3 (5 µM) or DMSO (0.1%) for 24 h. Western blotting was performed to assess the expression of TRKA and EGFR in cell lysates immunoprecipitated with an anti‐GRB2 antibody a). Quantification of TRKA levels b) and EGFR levels (c). d) SH‐SY5Y cells were treated with Rg3 (5 µM) or DMSO (0.1%) for 24 h after transfection with Scramble or TRKA siRNAs for 24 h. Quantification of mitochondrial CL in SH‐SY5Y cells. Three independent experiments per condition were performed in (a–d. e) The binding conformation of GRB2 and TRKA is depicted on the left. The binding conformation of GRB2 and TRKA after binding to Rg3 is depicted on the right. f–h) SH‐SY5Y cells were treated with Rg3 (5 µM), GW441756 (10 µM) or DMSO (0.1%) for 6 h. Analysis of higher‐molecular‐mass species containing GRB2 and TRKA was conducted via Native PAGE and immunoblotting, with GAPDH serving as the loading control (f). Representative images of western blotting of p‐TRKA, TRKA and GAPDH in cell lysates (g). Quantification of p‐TRKA levels (h). Three independent experiments per condition were performed in f–h. i–k) Wild‐type or mutant GRB2 was preincubated with Rg3 for 30 min. The binding affinity between TRKA and GRB2 (i), TRKA and GRB2‐Rg3 (j) and TRKA and GRB2 mutant‐Rg3 (k) was determined using a ForteBio Octet system. Three independent experiments per condition were performed in (i–k). Data are normalized to DMSO group (b, c and h). Mean ± standard error of the mean is presented. *p < 0.05, **p < 0.01, ***p < 0.001, ns, not significant. Student's two‐tailed unpaired t‐test (b, c), two‐way ANOVA with Sidak's multiple comparisons test (d), One‐way ANOVA with Tukey's multiple comparisons test (h). Source data are provided in the Source Data file.

Given that GRB2 directly binds to NTRK1 or TRKA^[^ ^22^ ^]^ and is essential for epidermal growth factor receptor (EGFR) signaling,^[^ ^38^ ^]^ our investigation focused on determining whether Rg3 protects SH‐SY5Y cells in a TRKA‐ or EGFR‐dependent manner. Treatment with Rg3 promoted the interaction between GRB2 and TRKA (**Figure** **3**a,b) with no discernible impact on the interaction between GRB2 and EGFR (Figure 3a,c) in SH‐SY5Y cells. Direct quantification of mitochondrial CL revealed that Rg3 treatment reinstated the reduced levels of mitochondrial CL induced by A53T‐αSyn, an effect that disappeared when TRKA was silenced (Figure 3d). Molecular docking analysis brought to light that the interaction of Rg3 with GRB2 augmented the number of binding sites from 9 to 11 in the optimal binding mode of GRB2 and TRKA (Figure 3e, Table S4, Supporting Information). For the specific inhibition of TRKA, GW441756 was employed, a compound binding to the extracellular domain of TRKA, thereby impeding the binding of nerve growth factor (NGF) to TRKA (Figure S6a, Supporting Information).^[^ ^39^ ^]^ Native PAGE experiments demonstrated that Rg3 facilitated the formation of a higher molecular mass species (MW = 250–300 kDa) containing GRB2 (MW = 25 kDa) and TRKA (MW = 130 kDa), corresponding to the calculated combined masses of two TRKA molecules and one GRB2 molecule. Notably, the formation of this complex remained unaffected by GW441756 treatment (Figure 3f). Consistently, Rg3 was found to elevate the phosphorylation of TRKA, while GW441756 demonstrated no effect on these changes (Figure 3g,h). Furthermore, we examined the interaction among Rg3, GRB2 and TRKA in vitro. Pre‐incubation with GRB2 and Rg3 resulted in a 5‐fold increase in the binding between TRKA and GRB2 (Figure 3i,j). Mutations (R67A/R86A/L120A) in the binding pocket of GRB2, disrupting its binding to Rg3, slightly diminished its affinity for TRKA (*K_D_* = 1.32E‐07 M) (Figure S6b and c, Supporting Information). Pre‐incubation with Rg3 failed to augment the binding affinity between mutant GRB2 (R67A/R86A/L120A) and TRKA (*K_D_* = 1.46E‐07 M) (Figure 3k). Notably, our analysis revealed that the interaction strength between Rg3 and TRKA was significantly lower, thousands of times weaker, compared to that of TRKA1 and GRB2 (*K_D_* = 7.36E‐04 M) (Figure S6d, Supporting Information). GRB2 binds to the intracellular domain of TRKA, thereby initiating TRKA activation and fostering its phosphorylation. Consequently, we postulate that this mechanism underlies the activation of TRKA by Rg3 through GRB2, and it remains unaffected by GW441756. From these findings, we speculate that Rg3 plays a driving role by establishing bonds between TRKA and GRB2 independently of NGF ligand.

### 2.4 Rg3 Induced Upregulation of CRLS1 Expression in Human Neural Cells

**Figure 4:**
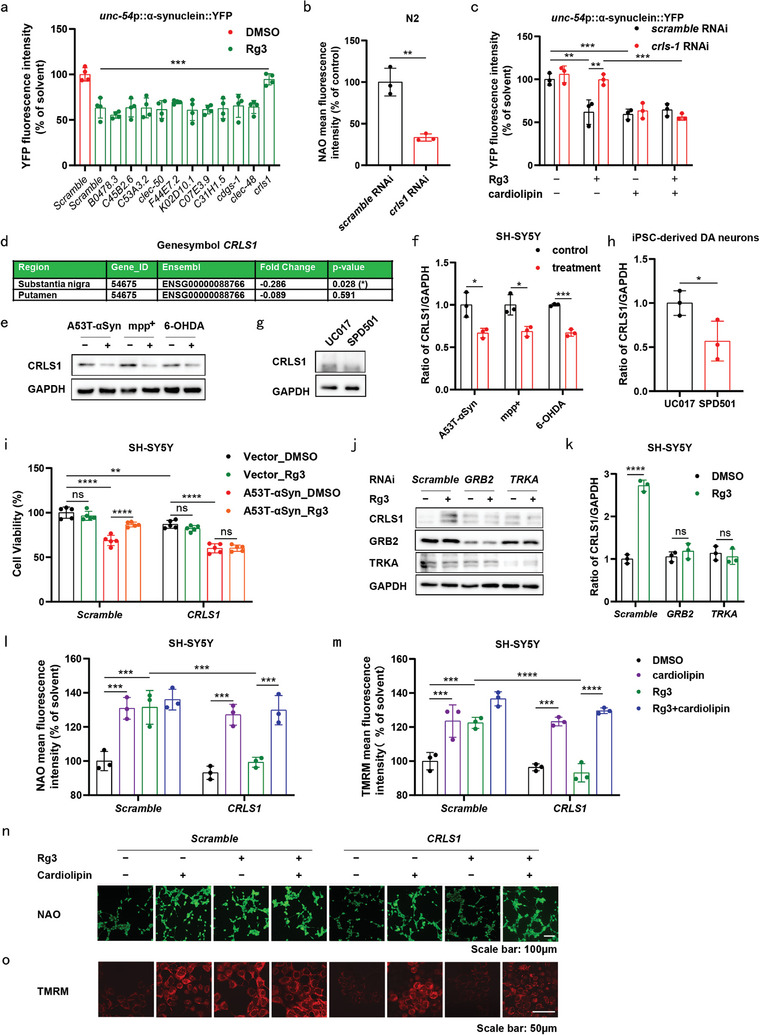
Rg3 induced upregulation of CRLS1 expression in human neural cells. a) YFP signals in NL5901 strains treated with Rg3 (10 µM) or DMSO for 12 days. Quantification of YFP fluorescence intensity is shown. Four independent experiments per condition were performed. b) N2 strains cultured for three generations on culture plates with bacteria containing indicated siRNAs. NAO staining evaluated CL levels in N2 strains. Quantification of mean NAO fluorescence intensity is shown. c) YFP signals in NL5901 strains treated with Rg3 (10 µM), CL (100 µg mL^−1^) or DMSO for 12 days. Quantification of YFP fluorescence intensity is shown. Three independent experiments per condition were performed in (b and c). d) Statistical analysis of genome‐wide differential expression analysis of *CRLS1* in the human SN and putamen. e–h) Representative western blot images of of CRLS1 in SH‐SY5Y cells transfected with pCMV3‐*Vector* or pCMV3‐*A53T‐αSyn*‐His for 48 h (e) or treated with mpp^+^ (600 µM) or 6‐OHDA (60 µM) for 24 h (e), and in UC017 and SPD501 DA neurons (g). Quantification of CRLS1 levels f,h). Three independent experiments per condition were performed in (e–h). i) SH‐SY5Y cells were treated with Rg3 (5 µM) or DMSO (0.1%) for 24 h after transfection with either *scramble* siRNA or *CRLS1* siRNA, and either pCMV3‐Vector or pCMV3‐A53T‐αSyn‐His for 24 h. Cytotoxicity was assessed using CCK‐8 assay, with five independent experiments per condition. j,k) Representative western blot images of CRLS1, GRB2, TRKA in SH‐SY5Y cells treated with Rg3 (5 µM) or DMSO (0.1%) for 24 h after transfection with Scramble, GRB2 or TRKA siRNAs for 24 h. Quantification of CRLS1 levels (k). l–o) SH‐SY5Y cells transfected with pCMV3‐*A53T‐αSyn*‐His, *scramble* siRNA or *CRLS1* siRNA for 24 h were treated with DMSO (0.1%), Rg3 (5 µM) or CL (10 µM) for 24 h. Quantification of mean NAO fluorescence intensity is shown (l). Quantification of mean TMRM fluorescence intensity is shown m). NAO staining for CL in SH‐SY5Y cells (scale bar = 100 µm) (n). TMRM staining was performed to assess mitochondrial membrane potential in SH‐SY5Y cells (scale bar = 50 µm) (o). Three independent experiments per condition were performed in (j–o). The representative images were obtained from three independent experiments (n, o). Data are normalized to Scramble group (b, c), DMSO group (f), UC017 group (h) or Scramble_DMSO group (i, k, l and m). Mean ± standard error of the mean is presented. *p < 0.05, **p < 0.01, ***p < 0.001, ****p < 0.0001, ns, not significant. One‐way ANOVA with Dunnett's multiple comparisons test (a), student's two‐tailed unpaired t‐test (b, f and h), two‐way ANOVA with Sidak's multiple comparisons test (c, i, k, l and m). Source data are provided in the Source Data file.

CL undergoes synthesis and metabolism through a cascade of enzymes (Figure S7a, Supporting Information). To identify the enzymes pivotal in maintaining CL levels in PD, we employed RNAi technology to silence each candidate. Strikingly, only knockdown of *CRLS1* resulted in the complete elimination of Rg3 effects in NL5901 worms (**Figure** **4**a). In line with previous findings, *CRLS1* knockdown reduced CL levels (Figure 4b; Figure S7b, Supporting Information), whereas CL replenishment reversed these effects in PD models (Figure 4c). These outcomes demonstrated that Rg3 elevated CL levels by activating CRLS1 to alleviate neuronal loss in PD models.

According to data from the Netherlands Brain Bank,^[^ ^40^ ^]^ a significant decline expression of CRLS1 gene was noted in SN between PD patients and control individuals (Figure 4d). Furthermore, a notable reduction in CRLS1 expression was observed in SH‐SY5Y cells transfected with A53T‐αSyn or subjected to mpp+ or 6‐OHDA treatment (Figure 4e,f). Protein expression of CRLS1 was also lower in SPD501 DA neurons compared to UC017 DA neurons (Figure 4g,h). The protective effects of Rg3 against A53T‐αSyn‐induced neural damage were completely abolished upon silencing of CRLS1 (Figure 4i). Notably, Rg3 treatment augmented CRLS1 expression in SH‐SY5Y cells, whereas knockdown of GRB2 or TRKA in these cells attenuated Rg3‐induced expression (Figure 4j,k). This implies that Rg3 activates CRLS1 expression through GRB2 and TRKA. Given the pivotal role of extracellular signal‐regulated kinase (ERK) in lipid synthesis,^[^ ^41^ ^]^ particularly as a downstream factor of TRKA^[^ ^42^ ^]^ and GRB2,^[^ ^43^ ^]^ we investigated ERK phosphorylation in SH‐SY5Y cells. Rg3 treatment enhanced ERK phosphorylation in SH‐SY5Y cells, but knockdown of GRB2 or TRKA in SH‐SY5Y cells attenuated Rg3‐induced ERK phosphorylation (Figure S7c–f, Supporting Information). This suggests that Rg3 activates ERK phosphorylation through GRB2 and TRKA. Importantly, treatment with the ERK‐specific inhibitor SCH772984^[^ ^44^ ^]^ eliminated the effects of Rg3 on CRLS1 (Figure S7g–i, Supporting Information).

Previous studies have reported the pivotal role of CL in preserving mitochondrial homeostasis. Thus, we probed whether Rg3 augments CL levels through CRLS1, contributing to the maintenance of mitochondrial homeostasis. Notable enhancements in CL levels and mitochondrial membrane potential were observed in A53T‐αSyn‐overexpressing SH‐SY5Y cells upon Rg3 treatment, as evidenced by NAO staining (Figure 4l and n) and TMRM staining (Figure 4m,o). Intriguingly, the silencing of *CRLS1* in these cells attenuated the Rg3‐induced rise in CL levels and mitochondrial membrane potential, a response reversible through CL supplementation (Figure 4l–o). Additionally, Rg3 facilitated the clearance of overexpressed A53T‐αSyn and the co‐localization of LC3 and mitochondria in SH‐SY5Y cells. However, the introduction of SCH772984 counteracted these effects (Figure S7j and k, Supporting Information), suggesting a role for Rg3 in promoting mitophagy to eliminate αSyn, contingent upon ERK signaling. In summary, our findings elucidate the activation of the TRKA–GRB2–ERK–CRLS1 pathway by Rg3, orchestrating the stimulation of CL biosynthesis for the preservation of mitochondrial homeostasis.

### 2.5 Rg3‐Mediated Upregulation of CRLS1 in an EVI1‐Dependent Manner

**Figure 5:**
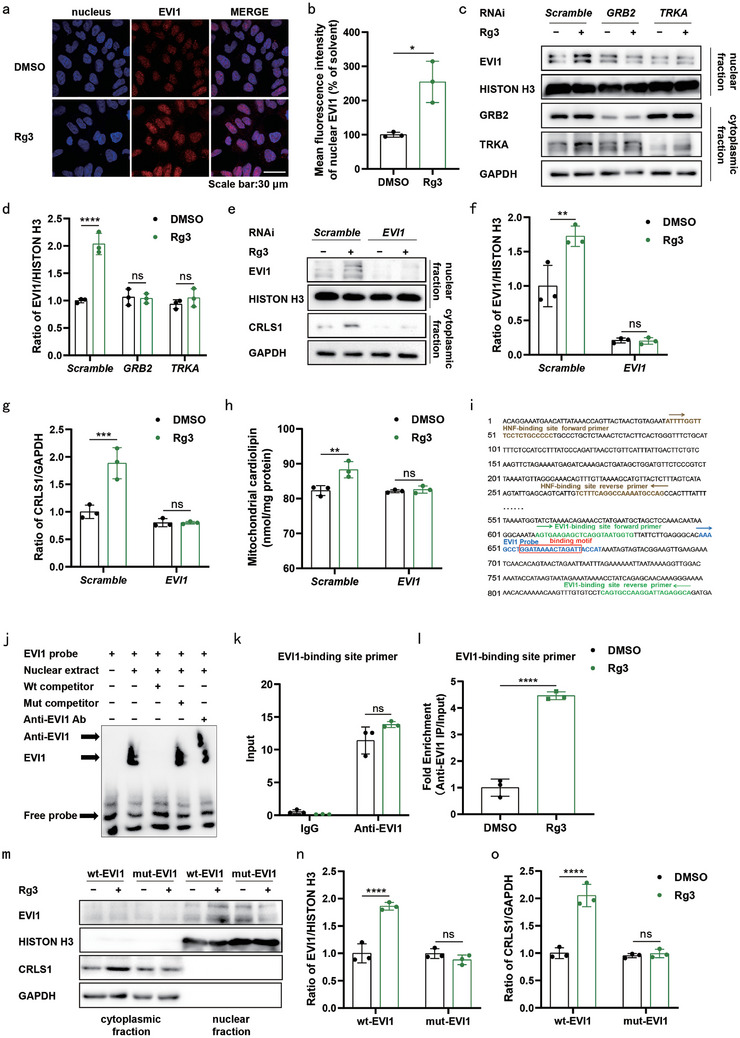
Rg3‐mediated upregulation of *CRLS1* in an EVI1‐dependent manner. a,b) SH‐SY5Y cells were treated with DMSO (0.1%) and Rg3 (5 µM) for 24 h. The cells were stained with antibodies against EVI1 (scale bar = 30 µm). Blue colour represents the nucleus a). Quantification of the mean fluorescence intensity of nuclear EVI1 is shown b). c,d) Nuclear and cytoplasmic protein fraction in SH‐SY5Y cells were treated with DMSO (0.1%), or Rg3 (5 µM) for 24 h after transfection with Scramble, GRB2 or TRKA siRNAs for 24 h. Representative western blot images of EVI1 in nuclear fraction and GRB2 and TRKA in cytoplasmic fraction are shown (c). Quantification of EVI1 levels (d). e–h) SH‐SY5Y cells were treated with DMSO (0.1%), or Rg3 (5 µM) for 24 h after transfection with Scramble or EVI1 siRNAs for 24 h. Representative western blot images of EVI1 in nuclear fraction and CRLS1 in cytoplasmic fraction are shown (e). Quantification of EVI1 levels (f) and CRLS1 levels (g). Quantification of mitochondrial CL in SH‐SY5Y cells (h). Three independent experiments per condition were performed in (a–h). The representative images were obtained from three independent experiments (a). i) Diagram of a putative EVI1‐binding site, a pair of EVI1‐binding site primers and a pair of HNF1A‐binding site primers in the CRLS1 promoter. j) The EVI1‐binding motif within the fragment of the CRLS1 promoter was verified via EMSA. k,l) ChIP assay revealed the enrichment of the CRLS1 promoter in DNA isolated from SH‐SY5Y cells treated with Rg3 (5 µM) and anti‐EVI1 antibodies. m–o) Following a 24 h transfection with either Scramble or EVI1 siRNAs, SH‐SY5Y cells were transfected with pcDNA3.1(+)‐EVI1 or pcDNA3.1(+)‐S728A‐EVI1 and treated with DMSO (0.1%) or Rg3 (5 µM) for 24 h. Representative western blot images of EVI1 in nuclear fraction and CRLS1 in cytoplasmic fraction are shown (m). Quantification of EVI1 levels (n) and CRLS1 levels (o). Three independent experiments per condition were performed in (j–o). Data are normalized to those of the DMSO group (b, l, n and o) or the Scramble_DMSO group (d, f, g and h). Mean ± standard error of the mean is presented. *p < 0.05, **p < 0.01, ***p < 0.001, ****p < 0.0001, ns, not significant. Student's two‐tailed unpaired t‐test (b, l), two‐way ANOVA with Sidak's multiple comparisons test (d, f, g, h, k, n and o). Source data are provided in the Source Data file.

To elucidate the mechanisms underlying the transcriptional upregulation of *CRLS1*, we predicted the transcription factors (TFs) and related TF‐binding sites using the match tool from TRANSFAC.^[^ ^45^ ^]^ Four putative TFs were identified in the promoter region of *CRLS1* (Table S5, Supporting Information). Each of these TFs was knocked down in SH‐SY5Y cells using RNAi technology (Figure S8a, Supporting Information). Notably, only *EVI1* knockdown eliminated the neuroprotective effects of Rg3 in the presence of A53T‐αSyn (Figure S8b, Supporting Information). Ecotropic Virus Integration Site 1 Protein Homolog (EVI1), encoding a critical oncogenic transcriptional regulator of hematopoietic stem cell proliferation,^[^ ^46^ ^]^ has been intensively investigated in leukemia and solid tumors.^[^ ^47^ ^]^ However, its involvement in neural system disorders remains enigmatic. In this study, immunofluorescence staining disclosed the predominant localization of EVI1 in the nucleus of SH‐SY5Y cells. Treatment with Rg3 significantly increased the expression of nuclear EVI1 (**Figure** **5**a,b). Similar results were observed in the nuclear fraction of SH‐SY5Y cells, but knockdown of GRB2 or TRKA in SH‐SY5Y cells attenuated Rg3‐induced EVI1 nuclear expression (Figure 5c,d). Upon *EVI1* knockdown, Rg3 neither promoted *CRLS1* expression (Figure 5e–g) nor increased mitochondrial CL levels (Figure 5h) in SH‐SY5Y cells. Validation of the binding of EVI1 to the promoter region of *CRLS1* was confirmed (Figure 5i), and an electrophoretic mobility shift assay (EMSA) demonstrated that the 655/668 region (GGATAAAACTAGATT) was the putative EVI1‐binding motif within the *CRLS1* promoter (Figure 5j; Table S6, Supporting Information). Chromatin immunoprecipitation (ChIP) assay revealed that EVI1 was recruited to the 609–845‐bp region containing the predicted EVI1‐binding motif (Figure 5k and l, Table S7, Supporting Information). Contrary to its effect on CRLS1, Rg3 exhibited no discernible impact on the expression of HNF1A, another predicted TF (Figure S8c and d, Supporting Information). These findings lend support to the notion that EVI1 functions as a specific TF governing *CRLS1* expression. Coimmunoprecipitation assay further revealed that Rg3 treatment enhanced the binding between EVI1 and ERK (Figure S8e and f, Supporting Information). In exploring the molecular intricacies, we identified the MAPK docking site, referred to as the DEF site (docking site for ERK FXF), in certain ERK substrates. Typically situated between 6 and 20 amino acids C‐terminal to the phosphorylation site, DEF sites have been implicated in ERK signaling pathways.^[^ ^48^ ^]^ Our speculation posits that the motif containing S728 serves as the putative target site for ERK (Table S8, Supporting Information). Subsequent interference with EVI1 expression in SH‐SY5Y cells revealed that Rg3 treatment prompted the nuclear translocation of wild‐type EVI1, while failing to induce a similar effect in the case of the mutant EVI1 (S728A) (Figure 5m–o; Figure S8g, Supporting Information). In synthesis, these results suggest that Rg3 orchestrates the activation of the TRKA–GRB2–ERK pathway, culminating in the nuclear translocation of EVI1 and the stimulation of *CRLS1* transcription.

### 2.6 Rg3 Alleviated A53T‐αSyn‐Induced Motor Function Deficits Through Grb2 and Crls1

**Figure 6:**
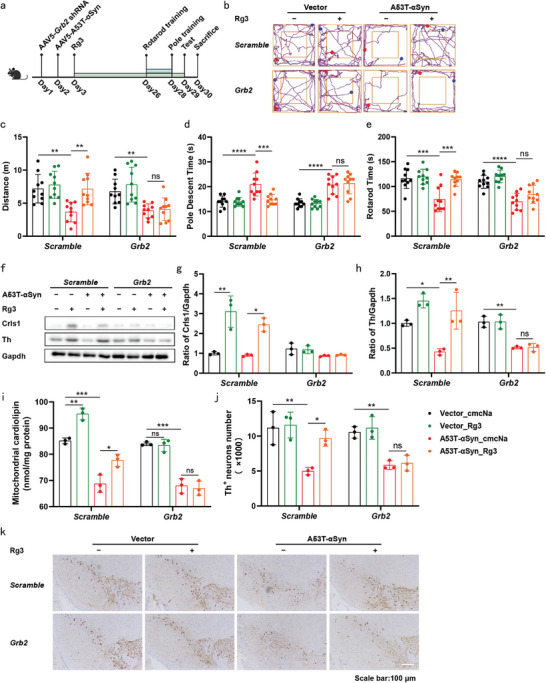
Rg3 alleviated A53T‐αSyn‐induced motor function deficits through Grb2 and Crls1. a) In vivo experimental scheme for assessing the therapeutic effects of Rg3. The brains and SN of mice were dissected for further analysis. b) Movement track in the open field test. c) Total distance travelled in the open field. d) Time required to descend the pole. e) Time taken to fall off the rotarod. (n = 10 mice per group in (b–e). f–h) Representative western blot images of Crls‐1, Th and Gapdh in SN (f). Quantification of Crls‐1 levels (g). Quantification of Th levels (h). i) Mitochondrial CL levels in the SN of mice. Three independent experiments were performed in (f–i). j) Quantification of TH^+^ neurons. k) Immunohistochemical staining of TH^+^ neurons (scale bar = 100 µm; n = 3 mice per group in (j, k). Data are normalized to Vector_cmcNa group (g,h). Mean ± standard error of the mean is presented. *p < 0.05, **p < 0.01, ***p < 0.001, ****p < 0.0001, ns, not significant. Two‐way ANOVA with Sidak's multiple comparisons test (c–e, g–j). Source data are provided in the Source Data file.

Motor dysfunction represents the primary pathological manifestation of PD in both human patients and murine models.^[^ ^49^ ^]^ This study delves into the investigation of Rg3's protective effects, utilizing two distinct mouse models of PD. The evaluation of motor function involved open field, rotarod, and pole tests subsequent to the final administration of Rg3 or carboxymethylcellulose sodium (cmcNa) in mice subjected to A53T‐αSyn or MPTP treatment (**Figure** **6**a; Figures S9a, S10a and S11a, Supporting Information).

Mice treated with A53T‐αSyn displayed a significant reduction in the total distance travelled over three minutes, indicating impairment of exercise ability. Notably, 20 mg kg^−1^ Rg3 treatment ameliorated this impairment, markedly enhancing the distance travelled by mice subjected to A53T‐αSyn (Figure S9b and c, Supporting Information). Moreover, mice subjected to A53T‐αSyn treatment exhibited prolonged descent times on the pole and quicker dismounts from the rotarod compared to their control counterparts. 20 mg kg^−1^ Rg3 treatment effectively reduced descent times (Figure S9d, Supporting Information) and significantly prolonged the time‐to‐fall from the rotarod compared to the cmcNa group (Figure S9e, Supporting Information). In mice subjected to A53T‐αSyn treatment, we observed that Rg3 exhibited dose‐dependent improvement in A53T‐αSyn pathology‐related motor deficits (1.25, 5 or 20 mg kg^−1^).

However, knockdown of *Grb2* or *Crls1* in the SN of mice, achieved through adeno‐associated virus serotype 5 (AAV5)^[^ ^50^ ^]^ transfection carrying *Grb2* shRNA or *Crls1* shRNA (Tables S2 and S3, Supporting Information), mitigated the beneficial effects of Rg3 on the motor function of mice (Figure 6b–e; Figures S10b–e and S11b–e, Supporting Information). Moreover, knockdown of *Crls1* resulted in diminished mitochondrial CL levels in the SN of mice, accompanied by impaired motor function and nigral DA neurons, which is consistent with the phenotype observed in neuron‐specific *Crls1* knockout mice (Figure S11b–e, Supporting Information).^[^ ^51^ ^]^ In accordance with the in vitro experiments, Rg3 treatment upregulated the Crls1 expression in the SN of mice (Figure 6f,g). Treatment with A53T‐αSyn or MPTP resulted in the depletion of mitochondrial CL in SN, whereas Rg3 treatment reversed these effects (Figure 6i; Figures S10f and S11f, Supporting Information). Furthermore, we evaluated the neuroprotective effects of Rg3 by comparing the abundance of nigral DA neurons between the treatment and non‐treatment groups. A53T‐αSyn treatment led to a severe loss of Th, which was reversed by Rg3 (Figure 6f,h). In addition, Rg3 intervention prevented the A53T‐αSyn‐ or MPTP‐induced depletion of nigral DA neurons (Figure 6j,k; Figures S10g and h, and S11g and h, Supporting Information). These observations imply that the improvements in motor function resulted from the protection of nigrostriatal DA neurons. Notably, the neuroprotective efficacy of Rg3 was entirely abrogated upon the knockdown of *Grb2* or *Crls1* in the SN of mice (Figure 6; Figures S10 and S11, Supporting Information).

In mammalian cells, the last step of CL synthesis is facilitated by CL synthase, where phosphatidylglycerol (PG) reacts with cytidine diphosphate‐diacylglycerol to form premature CL. Subsequently, the acyl chains undergo remodeling to yield mature CL.^[^ ^52^ ^]^ Notably, CL in mammalian brain tissue features a varied acyl chain composition enriched in long‐chain fatty acids (20:4 and 22:6), setting it apart from tissues such as the heart, skeletal muscle, and liver.^[^ ^53^ ^]^ Subsequently, we examined the effect of Rg3 on CL composition in the SN of A53T‐αSyn‐induced PD mice using liquid chromatography‐mass spectrometry (LC**/**MS) lipidomics analysis (Figure S12a and b, Supporting Information).^[^ ^54^ ^]^ Our results demonstrated that Rg3 treatment increased CL levels in the SN of both normal and A53T‐αSyn‐induced PD mice. Knockdown of *Grb2* or *Crls1* in the mouse SN reversed the effect of Rg3 on CL levels (Figure S12c, Supporting Information). Importantly, our analysis revealed no apparent preference for specific fatty acid chain lengths. Furthermore, lipidomics analysis indicated a corresponding trend in PG levels utilized for CL synthesis (Figure S12d, Supporting Information). Altogether, these results suggest that Grb2 and Crls1 plays a pivotal role in mediating the protective effects of Rg3 on nigral DA neurons in mice.

### 2.7 Synergistic Efficacy of Rg3 and L‐DOPA in Alleviating A53T‐αSyn‐Induced Motor Function Deficits

**Figure 7:**
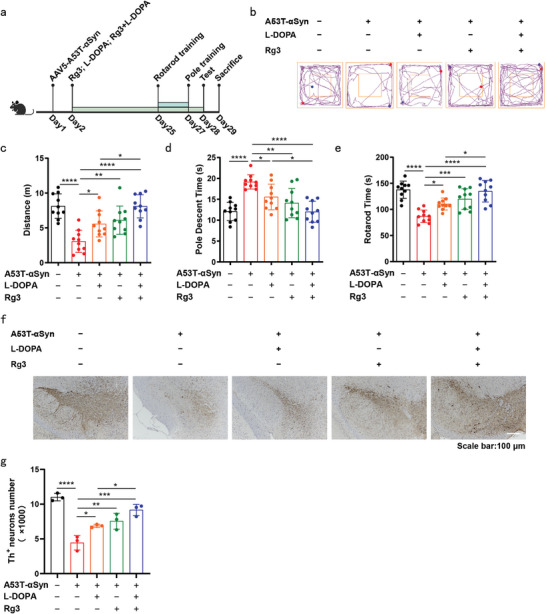
Synergistic efficacy of Rg3 and L‐DOPA in alleviating A53T‐αSyn‐Induced motor function deficits. a) In vivo experimental scheme for investigating the therapeutic effects of Rg3 (20 mg kg^−1^) and L‐DOPA (20 mg kg^−1^). The brain and SN of mice were dissected for further analysis. b) Movement track in the open field test. c) Total distance travelled in the open field test. d) Descent time in the pole test. e) Time‐to‐fall in the rotarod test (n = 10 mice per group in (b–e). f) Immunohistochemical staining of TH^+^ neurons (scale bar = 100 µm). g) Quantification of TH^+^ neurons (n = 3 mice per group in (f and g). Mean ± standard error of the mean is presented. *p < 0.05, **p < 0.01, ***p < 0.001, ****p < 0.0001. One‐way ANOVA with Tukey's multiple comparisons test (c–e, g). Source data are provided as a Source Data file.

Levodopa (L‐DOPA) is the standard medication prescribed to PD patients in clinical practice.^[^ ^55^ ^]^ However, prolonged L‐DOPA treatment is associated with potential motor function deterioration and the emergence of exercise ability.^[^ ^56^ ^]^ Upon entry into the brain, L‐DOPA undergoes conversion to dopamine in the striatum, thereby replenishing the depleted dopamine levels in PD patients.^[^ ^57^ ^]^ Given the differing mechanisms of action between Rg3 and L‐DOPA, we hypothesized that their concurrent administration might yield enhanced protective effects against motor dysfunction and neurodegeneration. To evaluate the protective effects of Rg3 and L‐DOPA, we administered A53T‐αSyn to mice and evaluated their motor function through open field, rotarod, and pole tests following the final treatment with Rg3 and L‐DOPA (**Figure** **7**a). Notably, L‐DOPA treatment resulted in increased distance travelled in the open field test, reduced descent time in the pole test, and prolonged time‐to‐fall in the rotarod test, indicative of improved motor function in A53T‐αSyn‐treated mice (Figure 7b–e). Moreover, L‐DOPA treatment prevented the loss of nigral DA neurons induced by A53T‐αSyn (Figure 7f,g). In comparison to L‐DOPA monotherapy, the combined administration of Rg3 and L‐DOPA exhibited significantly superior improvement in motor function and neuroprotective effects (Figure 7). These findings propose that the combination of Rg3 and L‐DOPA represents a novel and promising strategy for the clinical prevention of PD.

### 2.8 TDF Targeting the TRKA‐GRB2‐CRLS1 Axis Enhanced CL Expression and Alleviated Symptoms of PD

**Figure 8:**
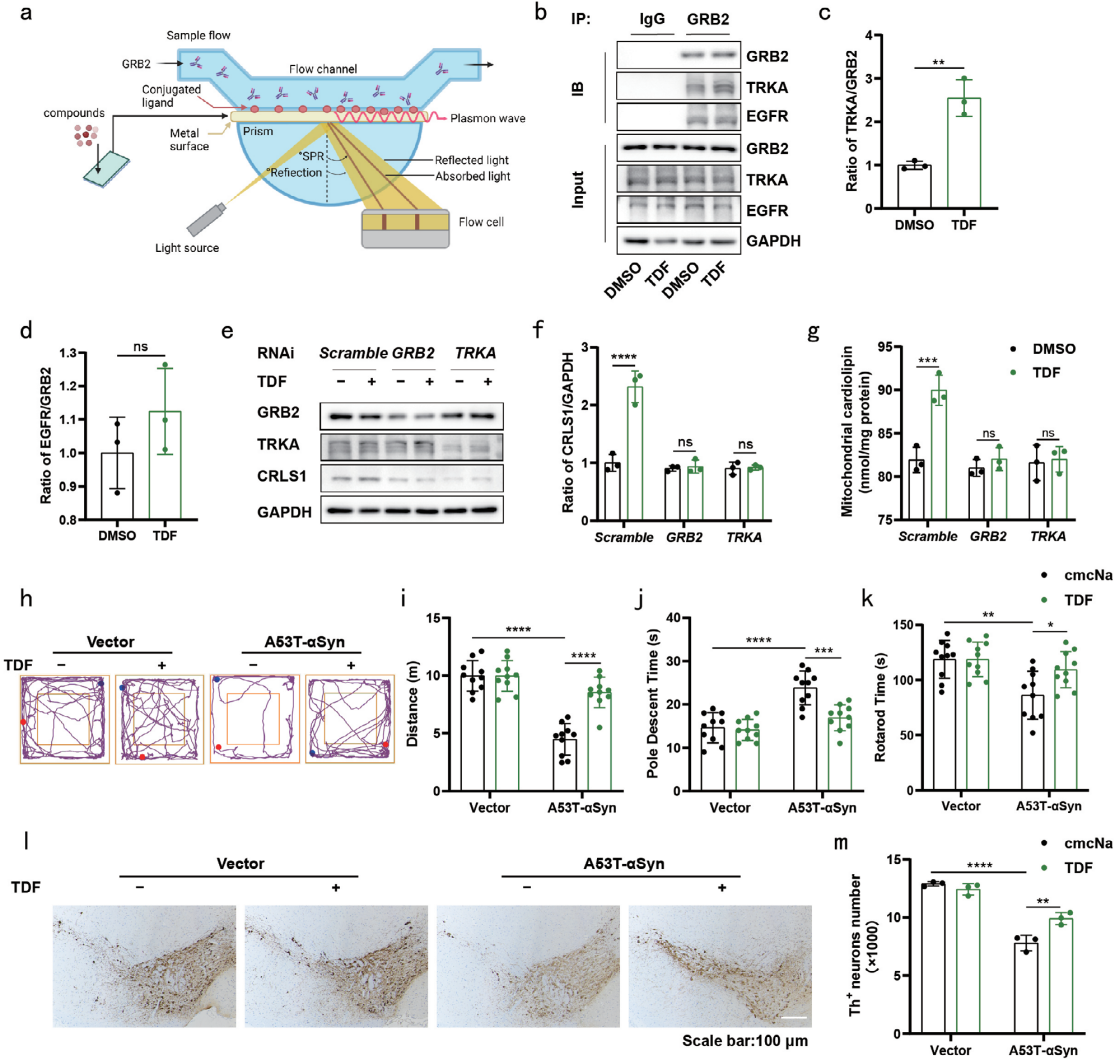
TDF targeting the TRKA‐GRB2‐CRLS1 axis enhanced CL expression and alleviated symptoms of PD. a) Experimental scheme for SPR technology to screen compounds binding to GRB2. b–d) SH‐SY5Y cells were stimulated with TDF (10 µM) or DMSO (0.1%) for 24 h. Western blotting was performed to evaluate the expression of TRKA and EGFR in cell lysates immunoprecipitated with an anti‐GRB2 antibody (b). Quantification of TRKA levels (c) and EGFR levels (d). e,f) Representative western blot images of CRLS1, GRB2, TRKA in SH‐SY5Y cells treated with TDF (10 µM) or DMSO (0.1%) for 24 h after transfection with Scramble, GRB2 or TRKA siRNAs for 24 h. Quantification of CRLS1 levels (f). g) SH‐SY5Y cells were treated with TDF (10 µM) or DMSO (0.1%) for 24 h after transfection with Scramble, GRB2 or TRKA siRNAs for 24 h. Quantification of mitochondrial CL in SH‐SY5Y cells. Three independent experiments per condition were performed in (b–g). h) Movement track in the open field test. i) Total distance travelled in the open field. j) Time required to descend the pole. k) Time required to fall off the rotarod. (n = 10 mice per group in (h–k). l) Immunohistochemical staining of TH^+^ neurons (scale bar = 100 µm). m) Quantification of TH^+^ neurons. (n = 3 mice per group in l, m). Data are normalised to those of the DMSO group (c,d) and the Scramble_DMSO group (f). Mean ± standard error of the mean is presented. *p < 0.05, **p < 0.01, ***p < 0.001, ****p < 0.0001, ns, not significant. Student's two‐tailed unpaired t‐test (c,d), two‐way ANOVA with Sidak's multiple comparisons test (f,g,i,j,k and m). Source data are provided in the Source Data file.

To further validate the universal impact of TRKA‐GRB2 axis‐targeted molecular in enhancing CL expression and ameliorating PD, we employed Surface Plasmon Resonance (SPR) technology^[^ ^58^ ^]^ to screen compounds that bind to GRB2 (**Figure** **8**a). Subsequently, we identified 11 compounds with a *K_D_* value at the microfriction level (Table S9, Supporting Information). The viability of SH‐SY5Y cells exposed to mpp+ or 6‐OHDA increased upon treatment with TDF and Clobetasone butyrate (Figure S13a and b, Supporting Information). TDF acts as a prodrug of tenofovir, a nucleotide reverse transcriptase inhibitor.^[^ ^59^ ^]^ On the other hand, Clobetasone butyrate is specially formulated for ophthalmological steroid‐based treatments.^[^ ^60^ ^]^ Considering its oral administration capability, we selected TDF for further research (Figure S13c and d, Supporting Information). TDF facilitated the clearance of overexpressed A53T‐αSyn in SH‐SY5Y cells (Figure S13e and f, Supporting Information) and promoted the interaction between GRB2 and TRKA without affecting the interaction between GRB2 and EGFR in these cells (Figure 8b–d). Treatment with TDF enhanced CRLS1 expression and restored the decreased mitochondrial CL levels caused by A53T‐αSyn in SH‐SY5Y cells. However, knockdown of GRB2 or TRKA in SH‐SY5Y cells attenuated TDF‐induced effect (Figure 8e–g). Moreover, TDF increased TRKA phosphorylation, ERK phosphorylation, and CRLS1 expression, while GW441756 had no impact on these changes (Figure S13g–j, Supporting Information). When SCH772984 was employed, it blocked the ability of TDF to increase ERK phosphorylation and CRLS1 expression (Figure S13k–m, Supporting Information). The impairment of exercise ability was alleviated following TDF (80 mg kg^−1^) treatment, resulting in an increase in the distance travelled by A53T‐αSyn‐treated mice (Figure 8h,i). Correspondingly, TDF exhibited a reduction in descent time (Figure 8j) and a substantial extension in the duration before falling off the rotarod (Figure 8k) when compared to the cmcNa group. Additionally, administration with TDF effectively mitigated the loss of nigral DA neurons induced by A53T‐αSyn (Figure 8l,m).

## 3 Discussion

In recent years, clinical and experimental investigations involving both human subjects and animal models have yielded compelling evidence elucidating the correlation between aberrant CL metabolism and neurofunctional deficits.^[^ ^7^ ^]^ The present study, quantifying a reduction in mitochondrial CL content in both in vitro and in vivo models of PD, provides additional emphasis on the widespread occurrence of CL irregularities in diverse PD pathological contexts, thereby perturbing mitochondrial homeostasis (Figure 1). Utilizing nematode models for screening, we identified ginsenoside Rg3, characterized by enhanced CL expression and substantiated anti‐PD effects (Figure S2, Supporting Information). Although CL primarily resides in the inner membrane of normally functioning mitochondria, a significant portion becomes exposed on the mitochondrial surface during periods of mitochondrial stress, where it undergoes peroxidation and subsequent degradation.^[^ ^11^, ^14^ ^]^ CL has been implicated in the facilitation of mitochondrial membrane pore formation, which is critical for mitochondrial permeability, leading to phenomena such as mitochondrial swelling and the release of cytochrome c.^[^ ^3^, ^61^ ^]^ We hypothesize that CL involved in mitochondrial membrane pore formation predominantly originates from the externalized pool on the mitochondrial surface.^[^ ^12^, ^13^ ^]^ Our findings demonstrate that Rg3 elevates CL levels without a commensurate increase in CL externalization (Figure S3i–l, Supporting Information), thereby preserving mitochondrial stability. This finding underscores our hypothesis that the functional role of CL in maintaining mitochondrial integrity is closely tied to its specific distribution within mitochondria. Furthermore, Rg3 exhibits selective activation of mitoUPR (Figure S2g and h, Supporting Information), while exerting no discernible impact on endoplasmic reticulum unfolded protein response or heat shock response (data not shown). Depletion of the pivotal mitoUPR protein, hsp‐6, results in the loss of the neuroprotective effect associated with heightened CL levels in 6‐OHDA impaired nematode models (data not shown). This underscores the indispensable role of hsp‐6 in the mitoUPR regulated by CL, thereby emphasizing the necessity for further investigation into its regulatory mechanisms. Consequently, employing Rg3 as a molecular probe, our aim is to explore pivotal targets and signaling pathways capable of modulating CL content.

In this investigation, we employed the LiP‐SMap method to identify a set of proteins binding to Rg3, the majority of which have not been previously reported (Figure 2a). The functions of these proteins in the regulation of CL and PD pathology remain predominantly unexplored. Therefore, this information offers valuable insights for further elucidating the mechanisms underlying the anti‐PD functions governed by CL. It was observed that the anti‐PD and mitochondrial CL‐enhancing effects of Rg3 are contingent upon GRB2 (Figure 2b,c). GRB2 binds to the intracellular domain of TRKA, facilitating TRKA autophosphorylation. This effect is further potentiated upon Rg3 binding to GRB2 (Figure 2d–h and Figure 3a–c) and remains unaffected by the extracellular domain inhibitor GW441756 of TRKA (Figure 3f–h). Notably, when the binding site of Rg3 with GRB2 is mutated (R67A/R86A/L120A), the previously mentioned facilitative effect diminishes (Figure 3i–k; Figure S6b–d, Supporting Information). The expression of NGF, abundantly present in the human SN, experiences a marked decrease in both PD patients and PD animal models.^[^ ^62^ ^]^ However, the utilization of NGF in treating neurodegenerative conditions remains restricted due to associated side effects such as pain and weight loss.^[^ ^63^ ^]^ In contrast, Rg3 facilitates the formation of the TRKA–GRB2 complex, allowing signal transduction to activate ERK independently of NGF (Figure S7c–f, Supporting Information). Diverging from conventional molecular glues typically employed for target protein degradation,^[^ ^64^ ^]^ Rg3 and TDF manifest as extraordinary molecular glues that actively stimulate TRKA‐GRB2 interaction. To the best of our knowledge, this investigation offers first evidence elucidating molecular glues that activate a receptor tyrosine kinase.

In the context of CL metabolic pathways, a multitude of pivotal enzymes operate, and our hypothesis posits that Rg3 likely enhances CL content through the regulatory modulation of these enzymes (Figure S7a, Supporting Information). Utilizing nematode models, we have pinpointed CRLS1 as a pivotal target subject to regulation by Rg3 in the adjustment of CL levels (Figure 4b). Remarkably diminished expression of CRLS1 has been discerned in individuals afflicted with PD (Figure 4d). Moreover, our investigation has unveiled, for the inaugural time, EVI1 as a transcription factor governing CRLS1, binding specifically to the CRLS1 promoter region at 655/668 (Figure 5i). Rg3 orchestrates the nuclear expression of EVI1 through the TRKA‐GRB2‐ERK pathway (Figure 5c,d), where ERK engages in a direct interaction with EVI1 (Figure S8e and f, Supporting Information). However, the phosphorylation sites of EVI1 necessitate additional validation through the application of specific antibodies. The EVI1–CRLS1 pathway contributes to the neuroprotective effects of NGF/TRKA signaling, in addition to the cAMP response element‐binding protein.^[^ ^65^ ^]^

**Figure 9:**
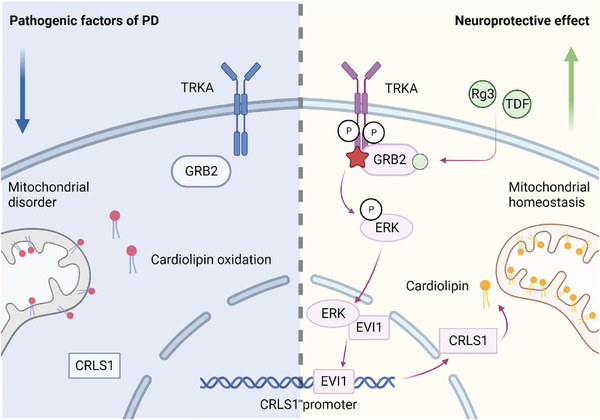
Schematic diagram of the mechanism through which Rg3 and TDF triggers the binding of GRB2 to TRKA and promotes ERK activation, resulting in EVI1 transactivation, *CRLS1* expression and CL biosynthesis.

In the current paradigm for PD treatment, the primary pharmacological intervention is L‐DOPA. This therapeutic agent operates by replenishing the dopamine precursor, thereby reinstating dopamine levels within the SN. In stark contrast to L‐DOPA, Rg3 manifests a discernibly distinct preventive mechanism in addressing PD. Notably, in murine models, the concurrent administration of Rg3 and L‐DOPA culminates in a more efficacious preventive outcome (Figure 7). The compound TDF, discerned through GRB2‐binding screening, shares a mechanistic similarity with Rg3 in fostering the expression of CL (Figure 8). Consequently, we posit that the TRKA‐GRB2‐EVI1‐CRLS1 signaling pathway could function as a screening metric to identify prospective pharmaceuticals targeting CL for PD prevention (**Figure** **9**).

## 4 Conclusion

We have elucidated the pathway that regulates the maintenance of cellular CL levels through controlling CRLS. The TRKA‐GRB2‐EVI1‐CRLS axis is crucial for the prevention of PD. In a groundbreaking discovery, we have identified two compounds, Rg3 and TDF, as a remarkable form of molecular glue adept at targeting and augmenting the interaction between GRB2 and TRKA, thus modulating this pathway and elevating mitochondrial CL levels. Through their pronounced neuroprotective effects, these compounds demonstrate promising potential for PD prevention. We believe that this work is of great interest not only to researchers in the field of neurodegenerative diseases but also carries far‐reaching implications for lipid metabolism and drug discovery.

## 5 Experimental Section

### Reagents

MPTP (M0896), mpp^+^ (D048), 6‐OHDA (H4381), cardiolipin (750332P) and NAO (A7847) were purchased from Sigma–Aldrich. Rg3 (HY‐N1376), L‐DOPA (HY‐N0304), SCH772984 (HY‐50846), GW441756 (HY‐18314), Tenofovir Disoproxil Fumarate (HY‐13782), and CCK8 (HY‐K0301) were purchased from Med Chem Express.

### Mice

All animal maintenance and experiment procedures adhered to national and international guidelines and received approval from China Pharmaceutical University, ensuring compliance with ethical standards for animal testing and research. Adult C57BL/6J mice were purchased from the China Yangzhou University, housed at temperatures ranging from 22 to 24 °C, maintained on a 12/12 h light/dark cycle, and provided with continuous access to food and water. In this study, animals were randomly grouped through the implementation of a method based on a random number table.

Three‐month‐old mice underwent stereotactic injection with either empty AAV5 (empty vector) or AAV5 expressing human mutated A53T‐aSyn (Vigene Biosciences). The 1.5 µL viruses, at a concentration of 10^12^ genomic particles/ml, were delivered into the right SN of mice at a rate of 0.5 µL min^−1^ using a microinjector.^[^ ^23^ ^]^ Injection coordinates from bregma were as follows: AP‐3.1 mm; ML‐1.4 mm; DV‐4.4 mm was applied for injection. Thirty days post A53T‐αSyn injection, the SN was dissected, and CL levels were assayed.

To induce a subacute MPTP‐induced PD model,^[^ ^24^ ^]^ three‐month‐old mice received intraperitoneal (i.p.) injection of 20 mg kg^−1^ MPTP dissolved in saline four times daily for seven consecutive days. Five days after the final MPTP treatment, the SN dissected and processed for CL level assessment.

In the knockdown group, AAV5‐*shGrb2* (10^12^ genomic particles/ml) was injected into the right SN of mice before A53T‐αSyn injection or MPTP administration. For A53T‐αSyn injection, mice in the treatment groups intragastrically (i.g.) administered 1.25 mg kg^−1^ Rg3, 5 mg kg^−1^ Rg3, 20 mg kg^−1^ Rg3,^[^ ^19^, ^66^ ^]^ 80 mg kg^−1^ TDF, 20 mg kg^−1^ L‐DOPA or a combined regimen of Rg3 and L‐DOPA daily, starting one day after A53T‐αSyn injection, and continued for 27 days. MPTP administration: In the treatment groups, mice were i.g. administered 20 mg kg^−1^ Rg3 daily, commencing on the first day of MPTP administration and continuing for 9 days. One day following motor function test, the mouse brain was dissected and processed for further experiments.

### Motor Function Tests

Three primary motor function tests were conducted to evaluate the efficacy of Rg3 alleviating motor function deficits in mice.

*Open field test*: Open field test was implemented to observe the locomotor activities of the animals. Locomotor activity was meticulously monitored using a camera integrated with the ANY‐Maze video‐tracking system (Stoelting Co., USA). The instrument captured the parameters associated with the locomotor activities of mice. Prior to the test, mice underwent a three‐minute training session inside the open field arena. Six hours post‐training, each mouse was gently placed in the center of the open field arena. Subsequently, data acquisition commenced through the software for the subsequent three minutes.

*Pole test*: This test involved a wooden pole measuring fifty centimeters in height and two centimeters in width. Mice were acclimatized for one day before the actual test. During the test, each mouse was positioned near the top of the pole, facing upwards. Time taken by each mouse to descend to the base of the pole was recorded as the pole descent time.

*Rotarod test*: Preceding the rotarod test, mice underwent a daily five‐minute placement on the rotarod instrument for three consecutive days to facilitate training. Following the training period, mice were positioned on the rotating rod, which increased in speed gradually from 0 to 40 rpm. Time taken by each mouse to slip from the rotating rod to the base of the instrument was recorded as the rotarod holding time.

### Immunohistochemistry

One day after motor function test, mice were euthanized, and their brains underwent processing for immunohistochemical studies. Tissue sections measuring 30 µm were subjected to a 24 h incubation period at 4 °C with a polyclonal anti‐tyrosine hydroxylase (Table S11, Supporting Information). HRP‐labeled Goat Anti‐Rabbit IgG(H+L) served as secondary antibodies and were applied to the sections for a 30 min incubation at room temperature. Nuclei were subsequently stained with hematoxylin at room temperature for 3 min. Utilizing Stereo Investigator software, images were captured, and the number of positive cells was quantified under microscope (Olympus BX51).

### Cell

Human neuroblastoma SH‐SY5Y cells (ATCC, CRL‐2266) were cultured in Dulbecco's Modified Eagle Medium (DMEM, Gibco, 11 965 092) supplemented with 10% fetal bovine serum (FBS, Gibco, A5669401). To induce the expression of A53T‐αSyn, SH‐SY5Y cells were transfected with pCMV3‐*A53T‐αSyn*‐His (Corues Biotechnology) and incubated for 2 days. In the establishment of an in vitro PD model, SH‐SY5Y cells were subjected to treatment with mpp^+^ (600 µM) or 6‐OHDA (60 µM) for 24 h. For overexpression of LC3 or TOM20, SH‐SY5Y cells were transfected with pCMV3‐*LC3*‐GFP and pCMV3‐*TOM20*‐RFP (Corues Biotechnology) and cultured for 2 days. Overexpression of S728A‐EVI1 was achieved by transfecting SH‐SY5Y cells with pcDNA3.1(+)‐*S728A‐EVI1* for 24 h, with pcDNA3.1(+)‐*EVI1* serving as the control. All plasmids were transfected into cells using lipofectamine 3000 (Thermofisher) according to the manufacturer's protocol. To downregulate the expression of specific genes in cells, siRNA targeting indicated gene (Table S2) was transfected using lipofectamine2000 (Thermofisher) following the manufacturer's protocol.

### iPSC‐Derived Dopaminergic Neurons

Stem cells were cultured in Floor Plate (FP) N1 medium consisting of 82% Knockout DMEM (Gibco), 15% Knockout serum replacement (Gibco), 1% GlutaMAX (Gibco), 1% NEAAs (Gibco) and 0.2% β‐mercaptoethanol (Sigma). The induction of FP‐cell‐based DA neurons followed established protocols. Upon reaching 80% confluency, iPSCs were disaggregated with accutase (STEMCELL) for 2 min, centrifuged at 200 × g for 3 min, plated on Matrigel‐coated multiwells at 2 × 105 cells cm^2^, and then incubated with FP cell induction medium N1 containing SB431542 (10 µM; Tocris) and LDN193189 (100 nM; Miltenyi Biotec). From day 1 to 7, SHH‐C24 (100 ng mL^−1^; Peprotech), FGF8 (100 ng mL^−1^; Peprotech), and purmorphamine (2 µM; Tocris) were introduced. CHIR99021 (3 µM; Tocris) was added from day 3 to 11. The transition from N1 to N2 medium commenced on day 5, with N1 and N2 mixed in ratios of 75% (N1): 25% (N2) on days 5 and 6, 50% (N1): 50% (N2) on days 7 and 8, and 25% (N1): 75% (N2) on days 9 and 10. On day 11, cells underwent passage for expansion and cryopreservation. N2 medium contained 98% Neural basal (Gibco), 1% N2 supplement and 1% GlutaMAX (Gibco). Following the sixth passage expansion, FP cells underwent disaggregation using accutase and were transferred onto a fresh Matrigel‐coated plate. Subsequently, the culture medium transitioned to DA induction medium in the presence of Y‐27632. The first stage of DA induction medium is composed of 96% NB (Gibco) and 2% B27 (Gibco), supplemented with GDNF (20 ng mL^−1^; Selleckchem), BDNF (20 ng mL^−1^; Peprotech), 0.2 mM ascorbic acid (AA, Sigma), DAPT (10 nM; Tocris), cAMP (10 µM; Sigma) and transforming growth factor β3 (1 ng mL). On day 8, cell dissociation using accutase facilitated their re‐plating onto dishes pre‐coated with 100ug/ml PDL (Sigma) and 5ug/ml laminin. Subsequently, the cells were sustained in the second stage differentiation medium (96% NB and 2% B27 supplemented with 20 ng mL^−1^ GDNF, 20 ng mL^−1^ BDNF, 0.2 mM AA, 10 nM DAPT, 20 µM cAMP) until achieving the desired maturation stage for a particular experimental context.

### Mitochondrial CL Levels Assay

Mitochondrial CL levels were quantified using the CL assay kit (Abcam, ab241036). Mitochondria were isolated from cells using the Mitochondria Isolation Kit (Beyotime Biotechnology, C3601) and from SN samples using the Mitochondria Isolation Kit (Beyotime Biotechnology, C3606). The protein concentration of isolated mitochondria was determined using the bicinchoninic acid method (Beyotime Biotechnology, P0009), ensuring a consistent protein level of 40 µg per group of isolated mitochondria. CL levels were measured on a multi‐label microplate reader (Envision) with the probe mix (48 µl of CL Buffer and 2 µl of CL Probe per well), and the reaction was monitored at 340/480 nm. CL levels were calculated based on a standard curve and expressed as nmol of CL per mg of protein.

### TMRM Staining

Cells underwent a single wash with phosphate‐buffered saline and were subsequently stained with TMRM Dye (5 µM), dissolved in phosphate‐buffered saline, at room temperature for 10 min in a dark room. Following staining, the samples underwent three washes with phosphate‐buffered saline and were directly observed using a confocal microscope (Olympus FV3000). Mean fluorescence intensity of TMRM was quantified using ImageJ software (NIH, USA).

### Cytochrome C Oxidase Activity Assay

Cytochrome c oxidase activity was assessed using Cytochrome c oxidase assay kit (Elabscience, E‐BC‐K837). Briefly, 2 × 107 cells were treated with the respective agents. Following incubation, cells were harvested and washed three times with cold PBS. Mitochondrial extraction from cell pellets was performed using the Mitochondria Isolation Kit for Cultured Cells (Beyotime Biotechnology, C3601). Subsequently, mitochondrial protein content was quantified, and 5 µg of mitochondrial protein was utilized for enzyme activity measurement. The enzymatic activity was determined on a multi‐label microplate reader (Envision) by monitoring the reduction of cytochrome c, measuring the absorbance decrease at 550 nm within the linear range of the reaction rate.

### ATP Assay

Intracellular ATP levels were quantified using the ATP chemiluminescence assay kit (Elabscience, E‐BC‐F002), following the manufacturer's instructions. Briefly, 2 × 10^7^ cells were treated with the specified agents, harvested, and washed thrice with cold PBS. Subsequently, the cells were lysed, and the resulting lysates were centrifuged at 10 000 g for 10 min at 4 °C. Chemiluminescent signals were detected at 636 nm using a multi‐label microplate reader (Envision).

### RNA Isolation and qRT‐PCR

Total RNA was extracted from iPSC‐derived DA neurons and SH‐SY5Y cells using TRIzol reagent (Invitrogen, 15 596 026) and then reverse transcribed into cDNA using HiScript II Q RT SuperMix (Vazyme, R222‐01). Real‐time PCR was performed in a 10 µl reaction system containing cDNA, primers, and SYBR (Vazyme, Q111) with 96‐well LightCycler real‐time PCR system (Roche). GAPDH served as an internal control gene. The 2^−ΔΔCt^ method was employed to calculate relative expression levels of mRNA. Primer sequences (Table S3, Supporting Information) were designed and synthesized by GenScript ProBio (Nanjing, China).

### CCK8 Assay

Cell viability was determined using the CCK‐8 assay. Cells were seeded at a density of 10^4^ cells per well in 96‐well plates. Following drug treatment, cells were incubated with 10% CCK‐8 dissolved in culture medium for 1 h. Absorbance at 450 nm was measured using SpectraMax iD5.

### C. *Elegans* Strains, Maintenance and RNAi

The *C. elegans* strains used in this study were obtained from the Caenorhabditis Genetics Center (University of Minnesota): Bristol (N2) strain as the wild‐type strain, NL5901 (pkIs2386 [*unc‐54*p:: α‐synuclein::YFP + unc‐119(+)]), BZ555 (egIs1 [*dat‐1*p::GFP]) and SJ4100 (zcIs13[*hsp‐6*p::GFP]). Worms were cultured at 20 °C on nematode growth media (NGM) agar plates, which were seeded with Escherichia coli (*E. coli*) strain OP50. Add compounds or CL to OP50 bacteria for 2–4 h. Then the bacteria were spotted on plates before transferring the worms.

RNAi experiments were performed by feeding worms *E. coli* strain HT115 (DE3), transformed with either the empty vector L4440 as the control or gene‐targeting constructs from the *C. elegans* Ahringer RNAi Collection. An overnight bacterial culture in Luria‐Bertani (LB), supplemented with Ampicillin (100 µg ml^−1^) at 37 °C, was seeded onto NGM plates containing isopropyl β‐D‐thiogalactoside (1 mM) and Ampicillin (100 µg ml^−1^). This was then incubated overnight at room temperature to induce the production of double‐stranded RNAs. Embryos or L1 larvae were placed on RNAi plates and incubated at 20 °C until adulthood for subsequent experiments.

### NAO Staining of Worms

N2 worms in the treatment groups were subjected to indicated compounds (10 µM), diluted in LB, for 36 h after pre‐treatment with 6‐OHDA (30 mM) dissolved in AA (10 mM) for 1 h at 20 °C. Following compounds treatment, 20–30 worms from each group were dislodged from plates with M9 for fixation. Briefly, worms were fixed with freshly prepared 0.5% paraformaldehyde and promptly frozen in liquid nitrogen. Subsequently, the worms underwent two freeze‐thaw cycles before complete thawing on ice and removal of the fixation solution. For NAO staining,^[^ ^28^ ^]^ a 10 µM of NAO solution was added for 30 min. Prior to imaging, worms were washed with M9 once to eliminate excess NAO from the solution. Images of fixed worms were acquired using an inverted fluorescence microscope (Nikon Ts2R) and quantified with ImageJ software (NIH, USA).

### Analyses of the Degeneration of DA Neurons

BZ555 worm was used for analysis of dopaminergic neurodegeneration.^[^ ^31^ ^]^ Worms in the treatment groups received indicated compounds (10 µM) diluted in LB for 72 h after pretreatment with 6‐OHDA (30 mM) dissolved in AA (10 mM) for 1 h at 20 °C. After fixation of BZ555 worms, GFP signals were measured by inverted fluorescence microscope (Nikon Ts2R) and quantified using ImageJ software (NIH, USA).

### Analyses of αSyn Aggregation

NL5901 worm was used for analysis of αSyn aggregation as described previously.^[^ ^32^ ^]^ In the treatment groups, worms were exposed to indicated compounds (10 µM), diluted in LB, for 12 days at 20 °C. Following fixation of NL5901 worms, YFP signals were measured by an inverted fluorescence microscope (BX53; (Nikon Ts2R) and quantified using ImageJ software (NIH, USA).

### Analyses of the mitoUPR of Worms

SJ4100 worms^[^ ^67^ ^]^ in the treatment groups received Rg3 (10 µM) diluted in LB for 72 h after pretreatment with 6‐OHDA (30 mM) dissolved in AA (10 mM) for 1 h at 20 °C. After fixation of SJ4100 worms, GFP signals were measured by inverted fluorescence microscope (Nikon Ts2R) and quantified using ImageJ software (NIH, USA).

### Lifespan Assay

Lifespan assay was performed on NL5901 worms treated with indicated compound at 20 °C.^[^ ^32^ ^]^ To prevent progeny production, 20 µg ml^−1^ (+)−5‐fluoro‐deoxyuridine was supplemented onto NGM plates during the reproductive period (Day 1 to 7 of adulthood). The initiation of adulthood was considered Day 1 on the survival curves. The worms were meticulously counted and categorized as alive, dead, or censored daily based on their movement and pharyngeal pumping. Kaplan‐Meier survival curves were plotted for each lifespan assay, and statistical analyses (log‐rank tests) were performed using GraphPad Prism 8.0.2 software.

### Western Blotting

Cell lysis was accomplished using RIPA lysis buffer (20 mM Tris‐HCl, 150 mM NaCl, 1% Triton X‐100, and 0.5% NP‐40) containing protease and phosphatase inhibitors, maintaining the samples on ice for 20 min. Similarly, SN tissues underwent lysis using RIPA lysis buffer containing protease and phosphatase inhibitors, undergoing three rounds of sonication over 30 min on ice. Then, extracted proteins were quantitated through the BCA method and subjected to denaturation by boiling at 99 °C for 20 min. Equal protein quantities were electrophoresed in 10% or 12% SDS‐PAGE and subsequently transferred onto a nitrocellulose membrane. The membrane was probed with primary antibodies overnight at 4 °C (Table S11, Supporting Information). The next day primary antibodies were removed, and the blots were washed with TBS buffer containing 0.1% Tween‐20. Corresponding secondary antibodies (1:1000, Beyotime) were applied at room temperature. Finally, blots were scanned with a chemiluminescence image analysis system (Tanon5200), and band intensities were quantified using ImageJ software (NIH, USA).

### NAO Staining of Cells

Following a 24 h incubation period with compounds, the cell supernatant was substituted with phenol red‐free medium containing 5 µM NAO for 20 min on ice in the dark. Subsequently, NAO was washed out three times with phenol red‐free medium. Fluorescence images were acquired using inverted fluorescence microscopy (Nikon Ts2R). Mean fluorescence intensity of NAO was quantified using ImageJ software (NIH, USA).

### CL Externalization Analysis

Cells were stained with 200 nM Mitotracker Red CMXRos (Thermofisher) for 30 min at 37 °C to label mitochondria prior to harvesting. Isolated crude mitochondria were incubated with FITC‐labeled Annexin V (Keygen Biotech, KGA1501) to stain for anionic phospholipids and then subjected to flow cytometric analysis (CytoFLEX S, BECKMAN COULTER) of the green FITC fluorescence.^[^ ^14a^ ^]^ The FITC fluorescence from gated far red, fluorescent mitochondria events was determined to evaluate the binding of Annexin V to mitochondria.

### Limited Proteolysis–Small‐Molecule Mapping

SH‐SY5Y cells underwent lysis using a lysis buffer (5% sodium deoxycholate, 1 mM KH2PO4, 3 mM Na2HPO4, 155 mM NaCl, pH 7.5) for 30 min at 4 °C. Following this, cell lysates were incubated with Rg3 (100 µM) or DMSO for 10 min at room temperature. These samples underwent limited proteolysis with proteinase K at room temperature for an additional 10 min to generate structure‐specific protein fragments. Subsequently, the fragments were subjected to trypsin protease digestion at 37 °C for 16 h, resulting in peptide mixtures suitable for subsequent proteomic analysis. The polypeptide samples were dissolved in 0.1% formic acid and detected by Orbitrap mass spectrometer, with label‐free quantitation analysis. Differential proteins were identified using the MaxQuant platform via MS sensitivity. Liquid phase conditions were as follows: column‐Analytical column (C18, 3 µm, 100 Å, 75 µm × 200 mm); Mobile phase‐A: 0.1% Formic acid; B: 0.1% acetonitrile formate; gradient‐ 2%–6% B 4 min, 9%–27% B 20 min, 33%–85% B 15 min, 90% B 10 min; flow velocity‐500 nl min^−1^. Mass spectrometry conditions: comprised a spray voltage of 2.8 kV, capillary temperature set at 350 °C; S‐lens at 65%, Collision energy at 30% HCD, resolution settings at one stage 60 000 m/z 200, two‐stage 15 000 m/z 200, parent ion scan range of m/z 300–1800, sub‐ion scanning range starting from m/z 110, data‐dependent MS/MS of top 20, isolation window of 1.6 Da, and a dynamic exclusion time of 40 s. The acquired MS/MS data were analyzed against a UniProtKB database by Andromeda algorithm built‐in MaxQuant engine (v1.5.5.0).

### Thermal Shift Assay

For the temperature‐dependent thermal shift assay, 150 µl of lysates (3 mg ml^−1^) derived from SH‐SY5Y cells were incubated with 5 µM of Rg3 or DMSO at each temperature point ranging from 37 to 60 °C for 3 min. Subsequently, the samples underwent repeated freezing and thawing using liquid nitrogen. Then, the samples were centrifuged at 20 000 g for 10 min at 4 °C to separate the supernatant and pellet. The supernatant was mixed with 5 × loading buffer and then separated on a 10% SDS‐PAGE for immunoblotting analysis targeting GRB2 and GAPDH.

### Expression and Purification of Protein

The human GRB2 and TRKA sequences were shown in Table S10 (Supporting Information). The pET‐28a‐GRB2 plasmid, pET‐28a‐GRB2(R67A/R86A/L120A) plasmid and pET‐28a‐TRKA plasmid were individually transformed into the Escherichia coli BL21(DE3) competent cells. Recombinant His‐tagged GRB2 protein, His‐tagged GRB2 (R67A/R86A/L120A) protein and His‐tagged TRKA protein were purified by chromatography on a Ni‐IDA ‐Sepharose CL‐6B column.

### ForteBio Octet System

The binding affinities between Rg3 and GRB2 or TRKA, as well as GRB2 and TRKA, were assessed using the fortebio octet system^[^ ^68^ ^]^ (ForteBio, CA, USA). Ni‐NTA sensors (Pall, no.18‐18‐5101) and Super Streptavidin (SSA) sensors (Pall, no.18‐5057) were preconditioned in Phosphate buffered saline‐Tween 20 (PBST) for 10 min before detection. For GRB2‐Rg3 binding affinity determination, either wild‐type or mutant GRB2 was loaded onto the Ni‐NTA sensors for 15 min. The processes of adsorption and desorption of the Rg3 molecule were concurrently monitored. After equilibrated with PBST for 3 min, the sensors were transferred into an Rg3 solution with three‐fold serial dilutions, ranging in concentrations from 2.44 to 66 µM. To evaluate TRKA‐GRB2 binding affinity, the biotin‐labeled reagent EZ‐Link NHS‐PEG12‐Biotin (Thermo, no.21312) was mixed with TRKA and allowed to react at room temperature for 30 min. The reaction was halted by removing excess biotin reagent using the PD MiniTrap G‐25 desalting column (GE, no. 28‐9180‐07). TRKA labeled with biotin was then loaded onto the SSA sensors for 15 min. After equilibrated with PBST for 3 min, the sensors were transferred into a solution of either wild‐type or mutant GRB2 with foure‐fold serial dilutions, ranging in concentrations from 20.8 to 1333 nM. For TRKA‐GRB2+Rg3 binding affinity, either wild‐type or mutant GRB2 was preincubated with Rg3 (20 µM) at room temperature for 30 min. TRKA labeled with biotin was loaded onto the SSA sensors for 15 min. After equilibrated with PBST for 3 min, the sensors were transferred into a solution of either wild‐type or mutant GRB2 with four‐fold serial dilutions, ranging in concentrations from 20.8 to 1333 nM. To determine TRKA‐Rg3 binding affinity, TRKA was loaded onto the Ni‐NTA sensors for 15 min. After equilibrated with PBST for 3 min, the sensors were transferred into an Rg3 solution with three‐fold serial dilutions, ranging in concentrations from 2.44 to 66 µM. Data analysis was conducted using Data Analysis HT 10.0 (ForteBio, CA, USA). The resulting binding curves were graphically represented using GraphPad Prism v8.0.2.

### Molecular Docking

The molecular docking calculations were performed using Discovery Studio 3.0 software. The 3D structure of the Rg3 was constructed and optimized via Sketching protocol in DS3.0. Crystal structure of TRKA (ID: 4AOJ) and GRB2 (ID: 1CJ1) were downloaded from protein data bank (https://www.rcsb.org/). These structures were independently preprocessed by deleting water molecules and adding hydrogen atoms for molecular docking studies. The LibDock protocol of DS3.0 was employed for semi‐flexible docking interactions between ligand (Rg3 or GRB2) and receptor (GRB2 or TRKA) in the CHARMm force field.

### Immunoprecipitation

Following the indicated compound incubation, cells were lysed in RIPA buffer. The cell homogenate underwent centrifugation at 17 500 × g for 15 min 4 °C, the supernatant was collected. Protein quantification was performed utilizing the BCA method. Subsequently, the cell lysate was immunoprecipitated with 2 µg of anti‐GRB2, anti‐ERK or normal IgG (Table S11, Supporting Information) overnight at 4 °C, followed by incubation with protein A‐agarose for 4 h at 4 °C. The formed Protein A‐agarose‐antigen‐antibody complexes were harvested via centrifugation at 10 000 × g for 1 min at 4 °C. Pellets were washed 3–4 times with 1 ml of IP buffer containing 20 mM Tris‐HCl (pH 8.0), 137 mM NaCl, 2 mM EDTA, 1% Nonidet P‐40, 10% glycerol, and protease inhibitor cocktails for 20 min each time at 4 °C. Bound proteins were resolved by SDS‐PAGE, followed by western blotting using anti‐GRB2, anti‐TRKA, anti‐ERK or anti‐EGFR antibodies (Table S11, Supporting Information). Additionally, western blotting included input samples from each.

### Native PAGE Analysis

Higher‐molecular‐mass complexes containing GRB2 and TRKA were detected by Native‐PAGE. SH‐SY5Y cell proteins were extracted using Native‐PAGE lysis buffer (50 mM Tris‐HCl, 150 mM NaCl, 1% NP‐40, and 0.25% sodium deoxycholate, pH 7.4), supplemented with protease and phosphatase inhibitors. After a 30 min lysis on ice, samples were centrifuged at 16 000 × g for 10 min, and the resulting supernatant was quantified. Samples were prepared with Native‐PAGE sample buffer and separated on a 4%–12% ExpressPlus PAGE Gel (GenScript) using native Tris‐mops running buffer (GenScript Biotech, M00727C) at 4 °C and 200 V. Proteins were subsequently transferred to a polyvinylidene fluoride membrane and processed for immunoblotting as described.

### Immunostaining

Cell samples were initially washed with PBS and subsequently fixed with 4% paraformaldehyde, followed by immunostaining. These samples underwent blocking with 2% BSA in PBS containing 0.5% Tween‐20 and 0.05% Triton X‐100 for 1 h. Then the samples were kept in primary antibodies for EVI1 (1:500) and incubated at 4 °C temperature overnight. The next day, PBS was employed for a 30 min washing period, after which the samples were subjected to further incubation with alexa fluor 555‐labeled donkey anti‐rabbit IgG (1:500, Beyotime) for 2 h. Following three 10 min washes with PBS, cells were incubated for 5 min with 4′,6‐diamidino‐2‐phenylindole (1:5000, Beyotime). Samples were observed by confocal microscope (Olympus FV3000). Mean fluorescence intensity of nuclear EVI1 were quantified using CellProfiler 4.2.5 software (Broad Institute, Britain).

### Prediction of Transcription Factor

To investigate gene regulation, it was focused on predicting the TFs that regulate CRLS1. Gene promoter sequences (upstream 2000 bp) were downloaded from UCSC (http://genome.ucsc.edu/). Subsequently, TFs and their associated TF binding sites (TFBS) were forecasted using the match tool from TRANSFAC (http://gene‐regulation.com/pub/databases.html), a repository providing information on eukaryotic transcription factors along with their experimentally validated binding sites (Table S5, Supporting Information).

### Electrophoretic Mobility Shift Assay

For DNA‐protein binding investigations, nuclear extracts from SH‐SY5Y cells were meticulously prepared. Biotin‐labeled wild‐type oligonucleotides and mutated oligonucleotides for EMSA probes were generously supplied by GENEWIZ (Suzhou, China) (Table S6, Supporting Information). DNA‐protein binding reactions were carried out using an EMSA/Gel‐Shift Kit (Beyotime, GS002) according to the manufacturer's instructions. Briefly, a 20 µL reactions contained 10 × binding Buffer (80% glycerin, 0.1 M MgCl2, 5 M NaCl, 1 M Tris‐HCl [pH 8.0], 0.5 M EDTA, 1 M DTT), ddH2O, probes, EVI1 antibody and nuclear extracts. Reactions are incubated at room temperature for 15 min, and subsequently, samples were loaded onto 10% ExpressPlus PAGE Gel (GenScript) after the addition of 5 µL loading buffer. Electrophoresis was performed for 45 min at 100 V in 0.5 × TBE buffer (0.045 mol L^−1^ Tris‐boric acid, 0.001 mol/L EDTA). Electro‐transfer was performed for 45 min at 380 mA with positively charged nylon membrane (Beyotime) in 0.5 × TBE buffer. After the transfer, the membranes underwent UV crosslinking of DNA with 254 nm wavelength at 120 mJ cm^2^ for 2 min. Immunoblots of the membranes were visualized by Chemiluminescent EMSA Kit (Beyotime, GS009).

### Chromatin Immunoprecipitation

ChIP assays were performed using a ChIP Assay Kit (Sigma‐Aldrich, 17–371). Briefly, SH‐SY5Y cells, subjected to a 24 h Rg3 treatment, underwent crosslinking with 1% formaldehyde for 20 min at 37 °C. The resultant cross‐linked adducts were resuspended and subjected to sonication. from the sonicated cell lysates was then immunoprecipitated utilizing the anti‐EVI1 antibody, followed by quantification using qRT‐PCR. Primers sequences were detailed in Table S7 (Supporting Information).

### LC/MS Based Lipidomic Profiling

LC**/**MS was performed as previously described.^[^ ^54^ ^]^ Briefly, the dried lipid extracts were dissolved in a mixed MS‐grade solution containing isopropanol, acetonitrile, water (2:1:1). Quality control (QC) samples were generated by combining equal volumes of each sample in the experiment. For quantitative analysis of lipids, 5 µL of samples was injected, with two QC samples were injected for every 10 samples. Chromatographic separation of lipids was performed on a C30 column (Acclaim C30 HPLC 5 µm, 100 × 4.6 mm), followed by detection using Orbitrap Exploris 240 mass spectrometer (Thermo Fisher) in negative ion mode. Liquid chromatography was configured as follows: buffer A: 600 ml acetonitrile, 399 ml water, 1 ml formic acid, and 0.631 g ammonium formate, Buffer B: 900 ml 2‐propanol, 99 ml acetonitrile, 1 ml formic acid, and 0.631 g ammonium formate. Flow rate was 0.26 mL mi^−1^n using the following method: T = 0 min, 30% B; T = 1 min, 55% B; T = 3 min, 65% B; T = 6 min, 85% B; T = 14 min, 100% B, T = 15 min, 100% B, T = 15.1 min, 30% B, T = 18 min, 30% B. Lipid identification was performed using Lipid Search 5.0 software. Initially, the extracted ion chromatogram (EIC) for each lipid parent ion was obtained for integration. Next, the product ion chromatogram from the EIC was extracted and deconvoluted. These chromatograms were then matched against a database. Finally, the results were appraised, and retention time alignment was carried out.

### Statistics

Statistics were performed using GraphPad Prism v8.0.2. Student's unpaired two‐tailed t‐test, one‐way ANOVA or two‐way ANOVA was conducted according to test requirements. *p < 0.05, **p < 0.01, ***p < 0.001 and ****p < 0.0001 were considered significant. The number of replicates and repeats of individual experiments and statistical tests was indicated in the legends.

### Ethics Approval

The animal study protocol was approved by the Institutional Animal Care and Use Committee of China Pharmaceutical University (Ethical approval number: 2023‐05‐022).

## Conflict of Interest

The authors declare no conflict of interest.

## Author Contributions

L.‐F.‐R.Q., S.L., and Q.F. contributed equally to this work. X.X. conceived the project. X.X. designed the experiments. L.‐F.‐R.Q., S.L., Q.F., C.Q., C.P., Y.L., M.Z., P.Y., P.W., and L.S. performed the experiments. Q.C. and L.C. provided bioinformatics and image analysis, respectively. L.‐F.‐R.Q., W.Y., P.L., and X.X. analyzed the data. L.‐F.‐R.Q., and X.X. wrote the paper with input from all authors.

## Supporting information

Supporting Information

Supplemental Tables

## Data Availability

The data that support the findings of this study are available in the supplementary material of this article.
