# Supplementary material for: Ginsenoside Rg3 Restores Mitochondrial Cardiolipin Homeostasis via GRB2 to Prevent Parkinson's Disease
Source: Adv Sci (Weinh). 2024 Aug 19;11(39):2403058. doi: 10.1002/advs.202403058 (PMC11497058; doi:10.1002/advs.202403058)
Supplement: Supplementary file 1 — Supporting Information [file ADVS-11-2403058-s001.docx]

**Supplementary information**


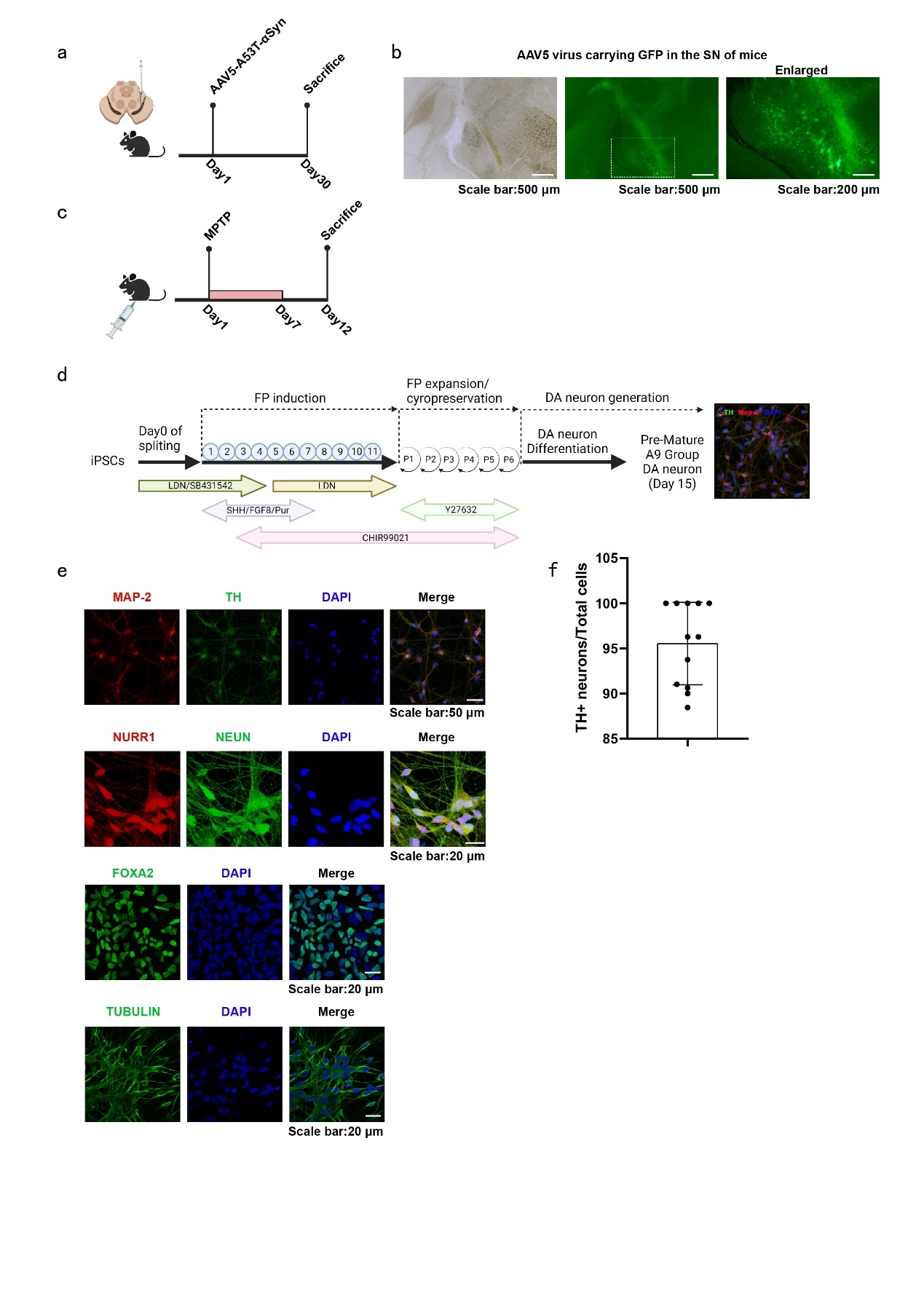


**Supplementary Figure 1**

**(a)** Experimental scheme for *in vivo* administration of AAV5-*Vector* and AAV5-*A53T-αSyn* in mice. **(b)** Representative fluorescence images of mouse midbrain sections. The GFP signal was detected in the ipsilateral SN following a 1-week interval post AAV5-GFP injection. **(c)** Experimental scheme for *in vivo* administration of saline and MPTP in mice. **(d**–**f)** Experimental scheme for the differentiation of DA neurons from iPSCs (**d**). Representative fluorescence images of TH, MAP-2, NURR1, NEUN, FOXA2 and TUBULIN in iPSC-derived DA neurons (**e**). Quantification of TH^+^ neurons (**f**).


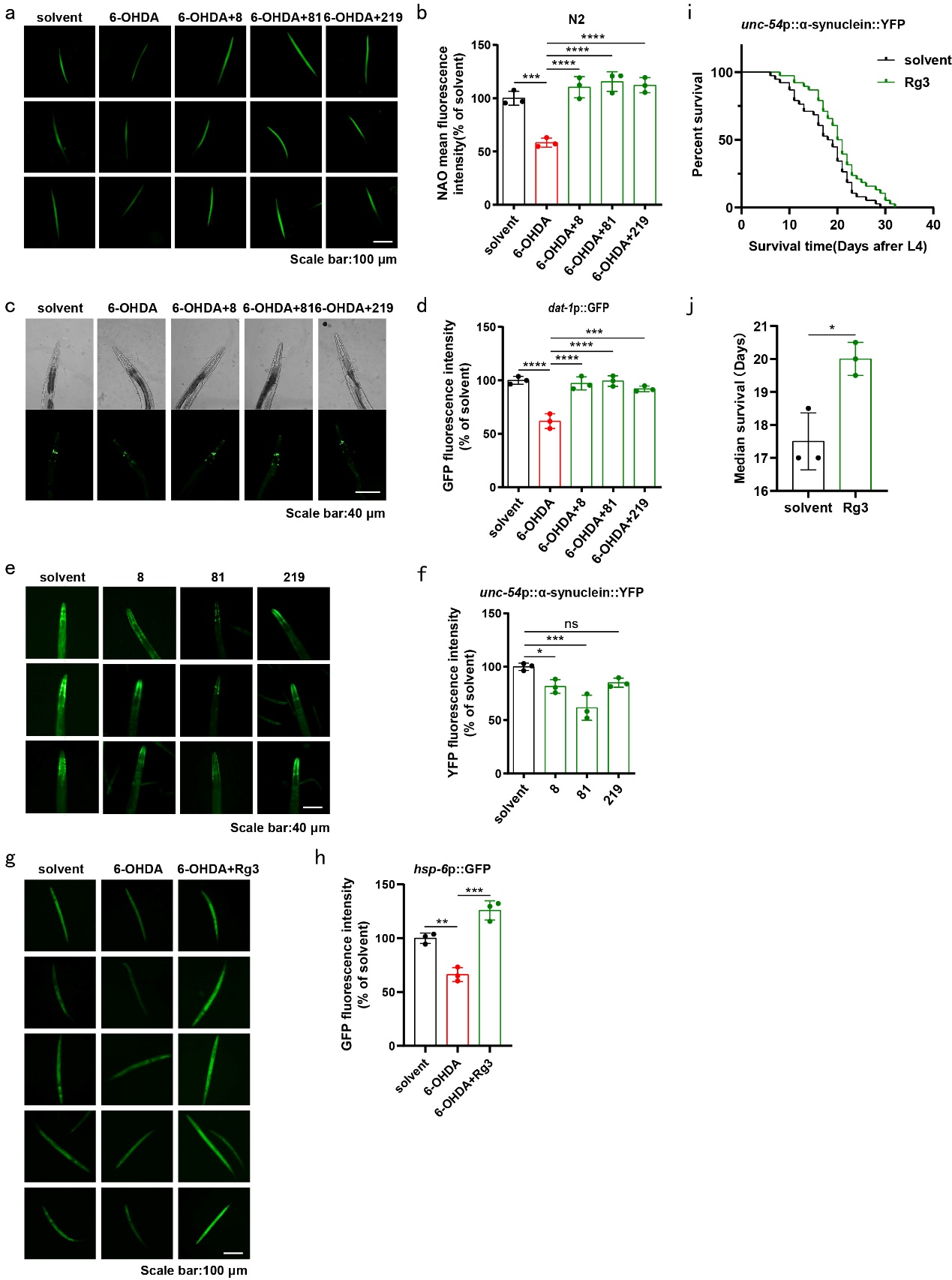


**Supplementary Figure 2**

**(a**, **b)** Evaluation of CL levels via NAO staining in the N2 strain treated with the corresponding natural products (10 µM) for 36 h after pretreatment with 6-OHDA (30 mM) for 1 h. Representative fluorescence images for each group are shown (scale bar = 100 µm) (**a**). Quantification of mean fluorescence intensity of NAO (**b**). **(c**, **d)** GFP signals in BZ555 worms treated with the corresponding natural products (10 µM) for 72 h after pretreatment with 6-OHDA (30 mM) for 1 h. Representative fluorescence images for each group are shown (scale bar = 40 µm) (**c**). Quantification of the GFP fluorescence intensity (**d**). **(e**, **f)** YFP signals in NL5901 strains treated with the corresponding natural products (10 µM) for 12 days. Representative fluorescence images for each group are shown (scale bar = 40 µm) (**e**). Quantification of YFP fluorescence intensity (**f**). **(g**, **h)** GFP signals in SJ4100 worms treated with the Rg3 (10 µM) for 72 h after pretreatment with 6-OHDA (30 mM) for 1 h. Representative fluorescence images for each group are shown (scale bar = 100 µm) (**g**). Quantification of the GFP fluorescence intensity (**h**). **(i**, **j)** Lifespan analysis of NL5901 strains treated with Rg3 (10 µM). Representative survival curves (**i**) and the median survival of NL5901 strains (**j**) are shown. Three independent experiments per condition were performed in a–j. Mean ± standard error of the mean is presented. *P < 0.05, **P < 0.01, ***P < 0.001, ****P < 0.0001, ns, not significant. One-way ANOVA with Tukey’s multiple comparisons test (**b**, **d** and **h**) or Dunnett's multiple comparisons test (**f**), Log-rank (Mantel-Cox) test (**i**), student’s two-tailed unpaired t-test (**j**). Source data are provided in the Source Data file.


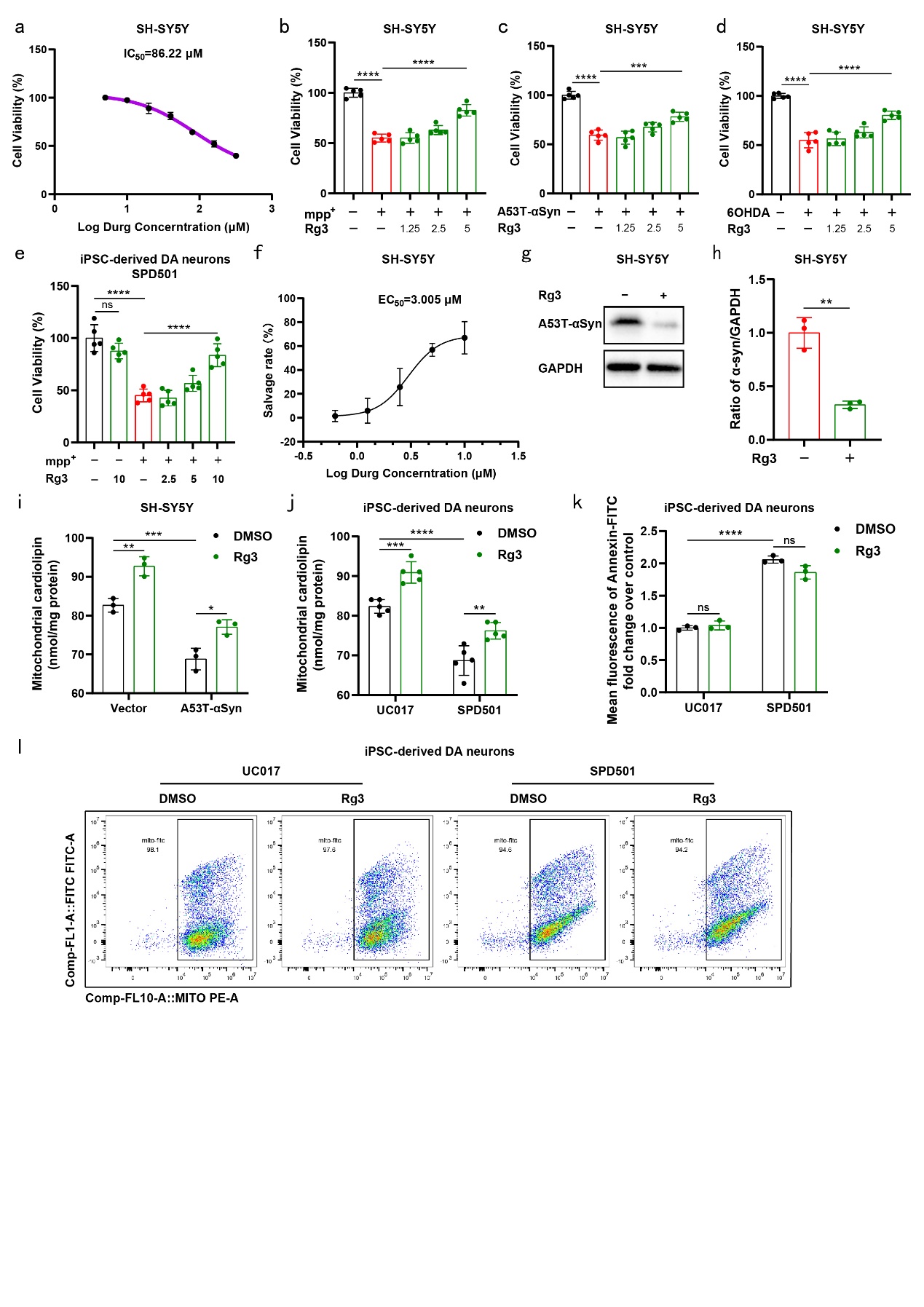


**Supplementary Figure 3**

**(a**–**f)** Cytotoxicity was evaluated via CCK-8 assay. Dehydrogenase activity in live cells of each group was quantified as a percentage relative to that in untreated cells. SH-SY5Y cells were treated with 5-, 10-, 20-, 40-, 80-, 160- or 320-μM Rg3 for 24 h. The IC_50_ value of Rg3 (**a**). Three independent experiments per condition were performed in a. SH-SY5Y cells were exposed to mpp^+^ (600 μM), Rg3 (1.25, 2.5 or 5 μM) or DMSO (0.1%) for 24 h (**b**). SH-SY5Y cells were treated with 1.25-, 2.5- or 5-μM Rg3 or DMSO (0.1%) for 24 h after pre-transfection with pCMV3-*Vector* or pCMV3-*A53T-αSyn*-His for 24 h (**c**). SH-SY5Y cells were treated with 6-OHDA (60 μM), Rg3 (1.25, 2.5 or 5 μM) or DMSO (0.1%) for 24 h (**d**). SPD501 DA neurons were exposed to mpp^+^ (600 μM), Rg3 (2.5, 5 or 10 μM) or DMSO (0.1%) for 24 h (**e**). Five independent experiments per condition were performed in b–e. SH-SY5Y cells were treated with mpp^+^ (600 μM), 0.625-, 1.25-, 2.5-, 5-, or 10-μM Rg3 for 24 h. The EC_50_ value of Rg3 (**f**). Three independent experiments per condition were performed in f. **(g**–**i)** SH-SY5Y cells were treated with Rg3 (5 μM) or DMSO (0.1%) for 24 h after pre-transfection with pCMV3-*Vector* or pCMV3-*A53T-αSyn*-His for 24 h. Representative images of western blotting of A53T-αSyn and GAPDH in SH-SY5Y cells are shown (**g**). Quantification of A53T-αSyn levels (**h**). Mitochondrial CL levels in SH-SY5Y cells (**i**). Three independent experiments per condition were performed in g-i. **(j**–**l)** UC017 and SPD501 DA neurons were treated with DMSO (0.1%) or Rg3 (10 μM) for 24 h. Mitochondrial CL levels were evaluated in UC017 and SPD501 neurons, with five independent experiments per condition (**j**). Evaluation of CL in the outer leaflet of OMM using Annexin V-binding assay. Cells were stained with Mitotracker Red CMXRos to label mitochondria prior to harvesting. Isolated mitochondria were incubated with FITC-labelled Annexin V to stain surface-exposed CL and then subjected to flow cytometric analysis (**k** and **l**). Three independent experiments per condition were performed in k and l. Data are normalized to DMSO group (**b**, **d**, **e**, **h** and **k**), Vector_DMSO group (**c**). Mean ± standard error of the mean is presented. **P < 0.01, ***P < 0.001, ****P < 0.0001. One-way ANOVA with Tukey’s multiple comparisons test (**b**–**e**), student’s two-tailed unpaired t-test (**h**), two-way ANOVA with Sidak’s multiple comparisons test (**i**–**k**). Source data are provided in the Source Data file.


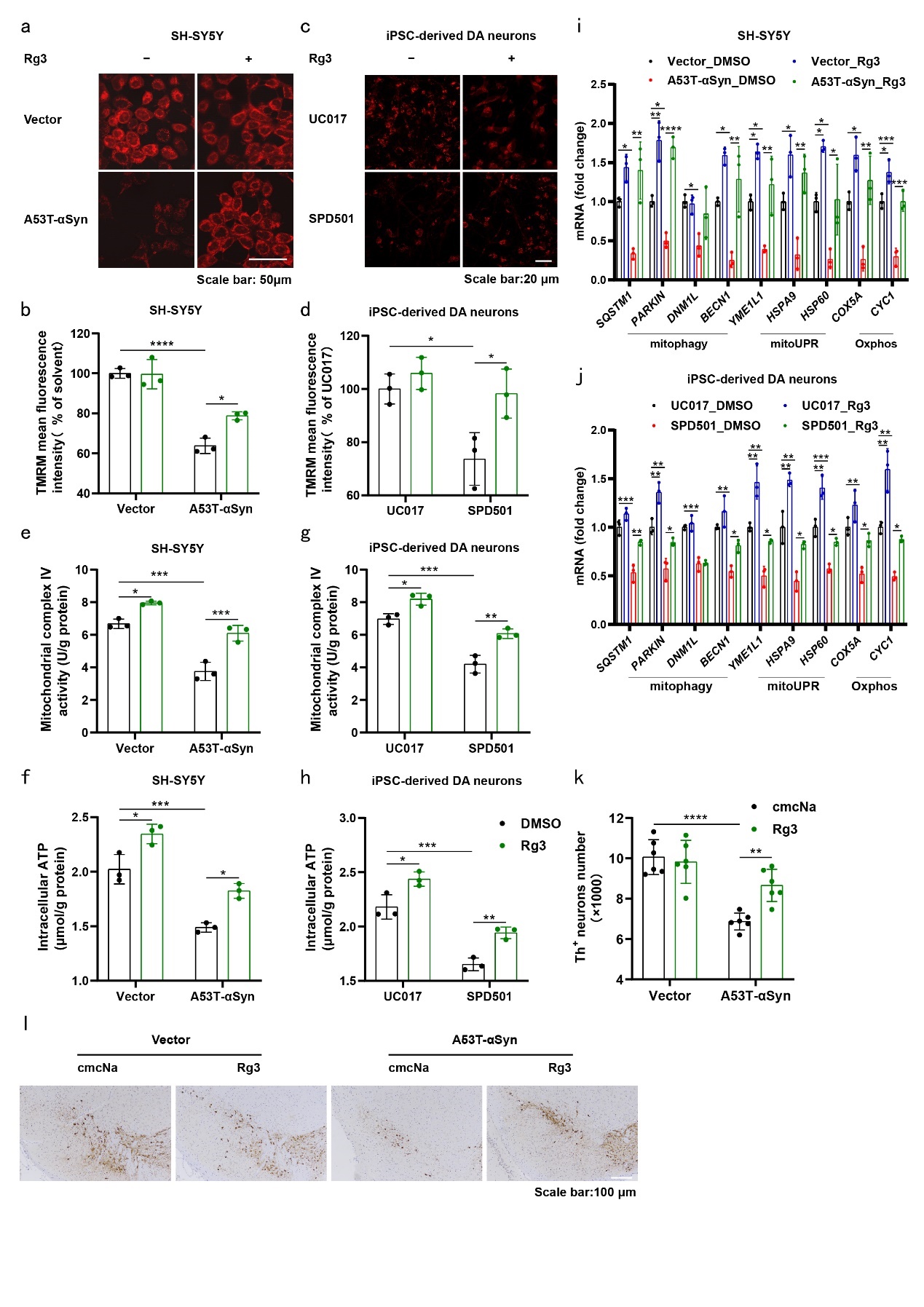


**Supplementary Figure 4**

SH-SY5Y cells were treated with Rg3 (5 μM) or DMSO (0.1%) for 24 h after pre-transfection with pCMV3-*Vector* or pCMV3-*A53T-αSyn*-His for 24 h (**a**, **b**, **e**, **f** and **i**). UC017 and SPD501 DA neurons were treated with DMSO (0.1%) or Rg3 (10 μM) for 24 h (**c**, **d**, **g**, **h** and **j**). **(a**, **b)** Mitochondrial membrane potential was assessed via TMRM staining in SH-SY5Y cells (scale bar = 50 µm) (**a**). Quantification of the mean fluorescence intensity of TMRM is shown (**b**). **(c**, **d)** Mitochondrial membrane potential was assessed via TMRM staining in UC017 and SPD501 DA neurons (scale bar = 20 µm) (**c**). Quantification of the mean fluorescence intensity of TMRM is shown (**d**). **(e**, **f)** Measurement of mitochondrial cytochrome c oxidase activity (**e**) and quantification of intracellular ATP concentration (**f**) in SH-SY5Y cells. **(g**, **h)** Measurement of mitochondrial cytochrome c oxidase activity **(g)** and quantification of intracellular ATP concentration **(h)** in UC017 and SPD501 DA neurons. **(i)** mRNA expression of genes related to mitophagy, mitoUPR and Oxphos in SH-SY5Y cells. **(j)** mRNA expression of genes related to mitophagy, mitoUPR and Oxphos in UC017 and SPD501 DA neurons. Three independent experiments per condition were performed in a–j. **(k**, **l)** AAV5-*Vector*-injected mice and AAV5-*A53T-αSyn*-injected mice received continuous oral administration of Rg3 (20 mg kg^−1^, i.g.) for 27 days per day. Quantification of TH^+^ neurons (**k**). Immunohistochemical staining of TH^+^ neurons (scale bar = 100 µm) (**l**). n = 6 mice per group in k and l. Data are normalized to Vector_DMSO group (**b**, **i**) and DMSO group (**d**, **j**). Mean ± standard error of the mean is presented. *P < 0.05, **P < 0.01, ***P < 0.001, ****P < 0.0001. Two-way ANOVA with Sidak’s multiple comparisons test (**b**, **d**–**k**). Source data are provided in the Source Data file.


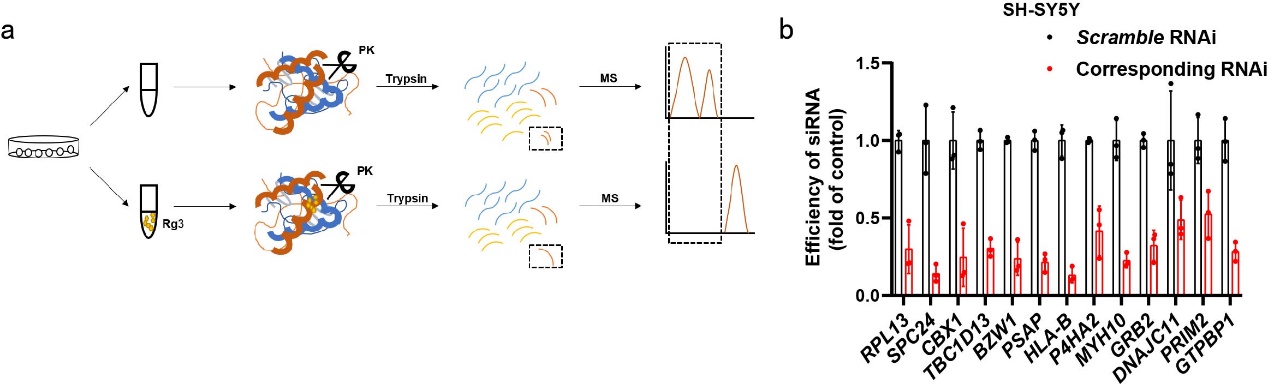


**Supplementary Figure 5**

**(a)** Experimental scheme for LiP-SMap. (**b)** Silencing efficiency of indicated siRNAs in SH-SY5Y cells. Data are normalised to those of the *scramble* group. Mean ± standard error of the mean is presented. Source data are provided in the Source Data file.


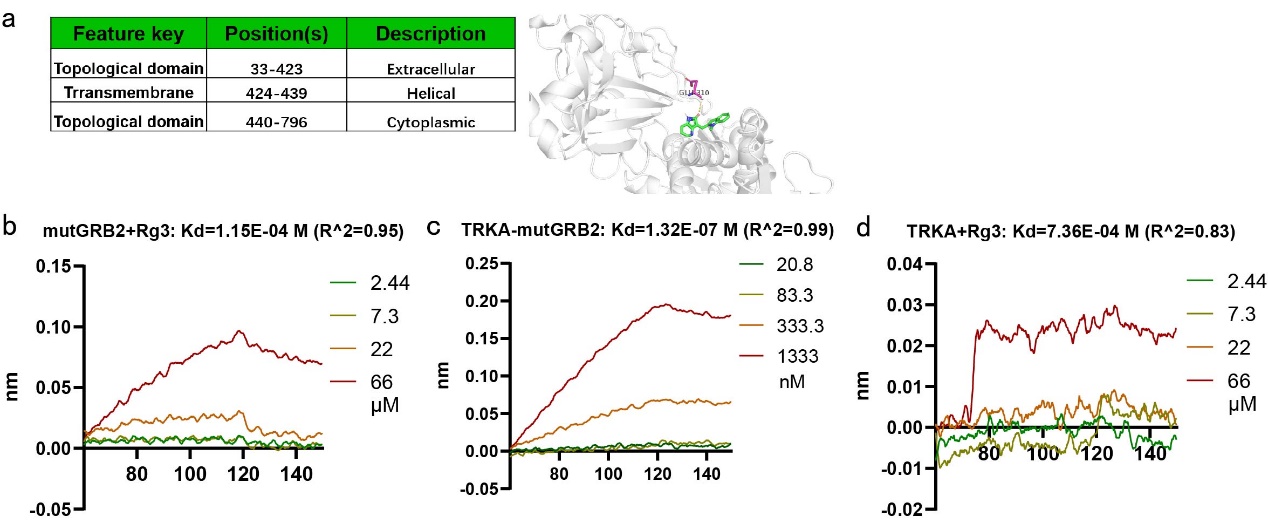


**Supplementary Figure 6**

**(a)** Positions of the extracellular, helical and cytoplasmic domains of TRKA. Glu-310 cross-linked with GW441756. **(b**–**d)** The binding affinity between mutant GRB2 and Rg3 (**b**), TRKA and Rg3 (**c**) and TRKA and mutant GRB2 (**d**) was determined on a ForteBio Octet system. Source data are provided in the Source Data file.


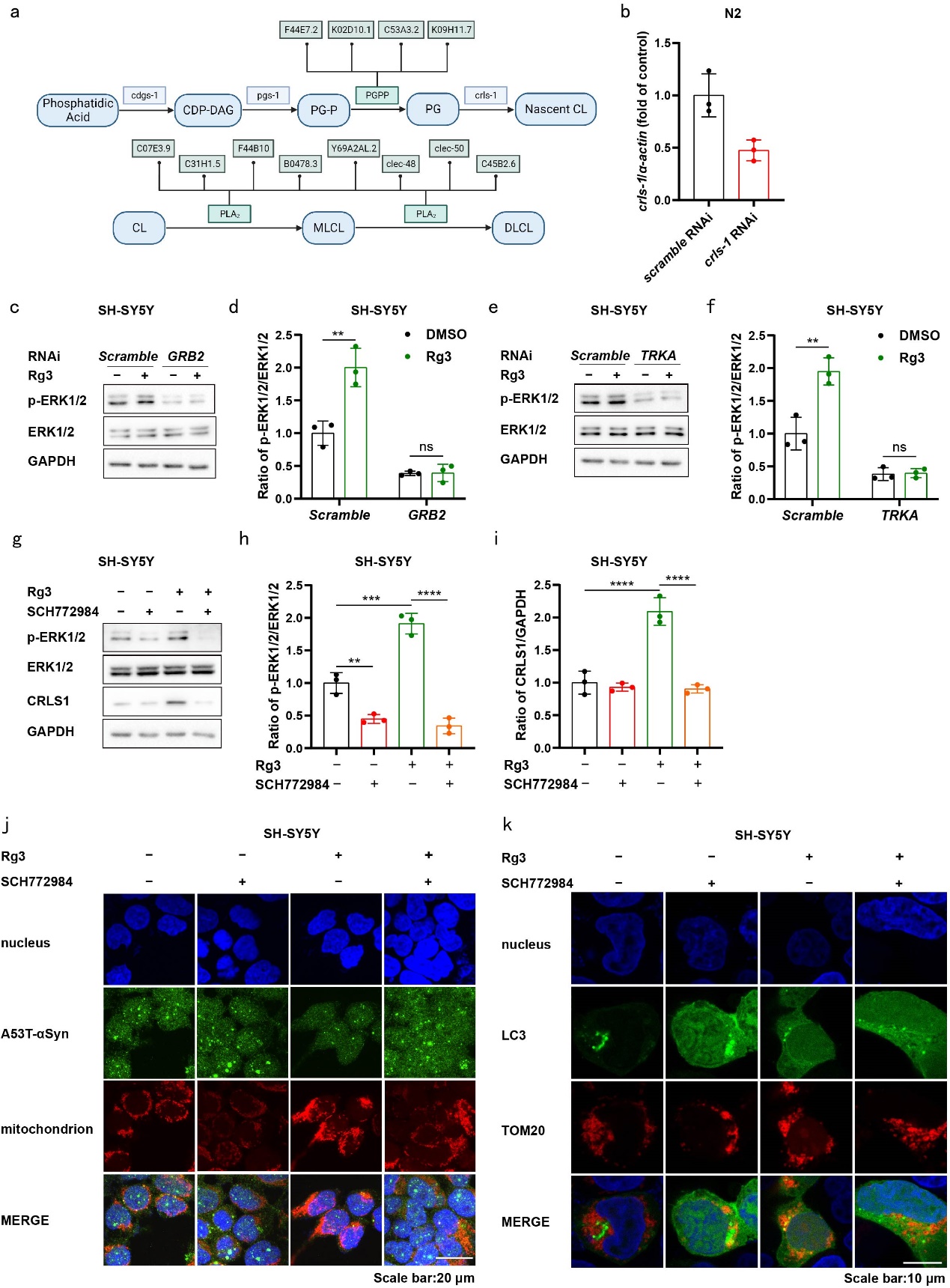


**Supplementary Figure 7**

**(a)** Genes involved in CL synthesis and hydrolysis. **(b)** N2 strains were cultured for three generations on culture plates with bacteria containing the indicated siRNAs. The mRNA expression of *Crls-1* was examined in N2 strains. **(c–f)** SH-SY5Y cells were treated with Rg3 (5 μM) or DMSO (0.1%) for 24 h after transfection with *scramble* siRNA, *GRB2* siRNA (**c**) or *TRKA* siRNA (**e**) for 24 h. Representative images of western blotting of p-ERK, ERK and GAPDH in cell lysates are shown (**c**, **e**). Quantification of p-ERK levels (**d**, **f**). **(g–i)** SH-SY5Y cells were treated with DMSO (0.1%), Rg3 (5 μM) or SCH772984 (10 μM) for 24 h. Representative images of western blotting of p-ERK, ERK, CRLS1 and GAPDH in cell lysates are shown (**g**). Quantification of p-ERK levels (**h**) and CRLS1 levels (**i**). **(j)** SH-SY5Y cells transfected with pCMV3-*A53T-αSyn*-His for 24 h were treated with DMSO (0.1%), Rg3 (5 μM) or SCH772984 (10 μM) for 24 h. Representative images of immunofluorescence staining of A53T-αSyn, DAPI (nucleus) and mitotracker (mitochondrion) signals in SH-SY5Y cells are shown (scale bar = 20 µm). **(k)** SH-SY5Y cells were transfected with pCMV3-*A53T-αSyn*-His, pCMV3-MAP1LC3A-GFP and pCMV3-TOM20-RFP for 24 h and treated with DMSO (0.1%), Rg3 (5 μM) or SCH772984 (10 μM) for 24 h. DAPI (nucleus), GFP and RFP signals were detected in SH-SY5Y cells (scale bar = 10 µm). Three independent experiments per condition were performed in b–i. The representative images are obtained from three independent experiments (**j**, **k**). Data are normalized to Scramble group (**b**), Scramble_DMSO group (**d**, **f**) or DMSO group (**h**, **i**). Mean ± standard error of the mean is presented. **P < 0.01, ***P < 0.001, ****P < 0.0001, ns, not significant. Two-way ANOVA with Sidak’s multiple comparisons test (**d**, **f**), one-way ANOVA with Tukey’s multiple comparisons test (**h**, **i**). Source data are provided in the Source Data file.


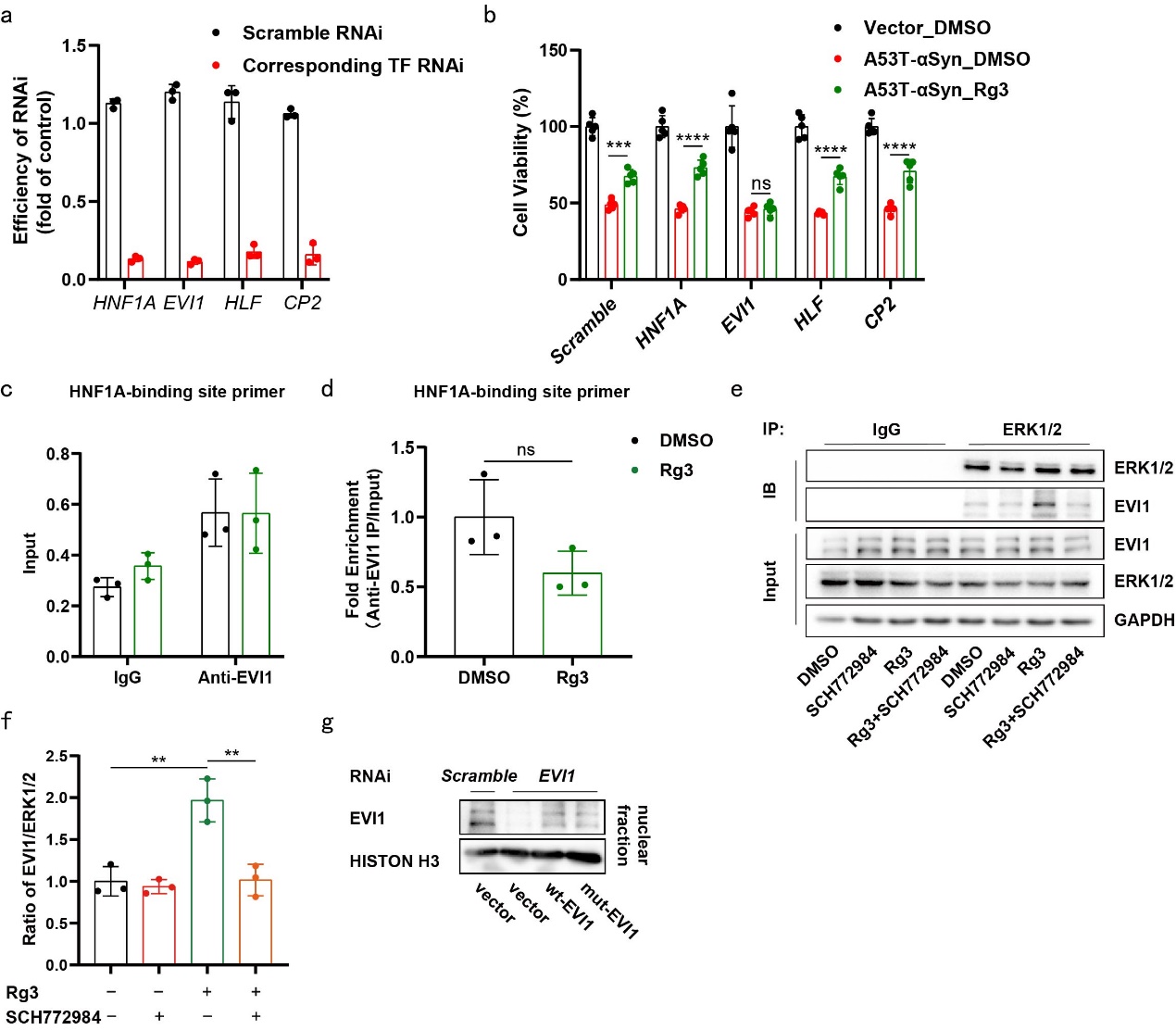


**Supplementary Figure 8**

**(a)** Silencing efficiency of the indicated siRNAs in SH-SY5Y cells, with three independent experiments per condition. **(b)** SH-SY5Y cells were treated with Rg3 (5 μM) or DMSO (0.1%) for 24 h after transfection with the indicated siRNAs and pCMV3-*Vector* or pCMV3-*A53T-αSyn*-His for 24 h. Cytotoxicity was evaluated via CCK-8 assay, with five independent experiments per condition. **(c**, **d)** ChIP assay revealed the enrichment of the CRLS1 promoter in DNA isolated from SH-SY5Y cells treated with Rg3 (5 μM) and anti-EVI1 antibodies. **(e**, **f)** SH-SY5Y cells were treated with DMSO (0.1%), Rg3 (5 μM) and SCH772984 (10 μM) for 24 h. Western blotting was performed to assess EVI1 expression in cell lysates immunoprecipitated with an anti-ERK antibody (**e**). Quantification of EVI1 levels (**f**). **(g)** After transfection with Scramble or EVI1 siRNAs for 24 h, SH-SY5Y cells were transfected with pcDNA3.1(+)-EVI1 or pcDNA3.1(+)-S728A-EVI1 for 24 h. Representative images of western blotting of EVI1 in nuclear fraction are shown. Three independent experiments per condition were performed. Data are normalized to Vector_DMSO group (**b**) or DMSO group (**d**, **f**). Mean ± standard error of the mean is presented. **P < 0.01, ***P < 0.001, ****P < 0.0001, ns, not significant. One-way ANOVA with Tukey's multiple comparisons test (**b**, **f**), two-way ANOVA with Sidak’s multiple comparisons test (**c**), student’s two-tailed unpaired t-test (**d**). Source data are provided in the Source Data file.


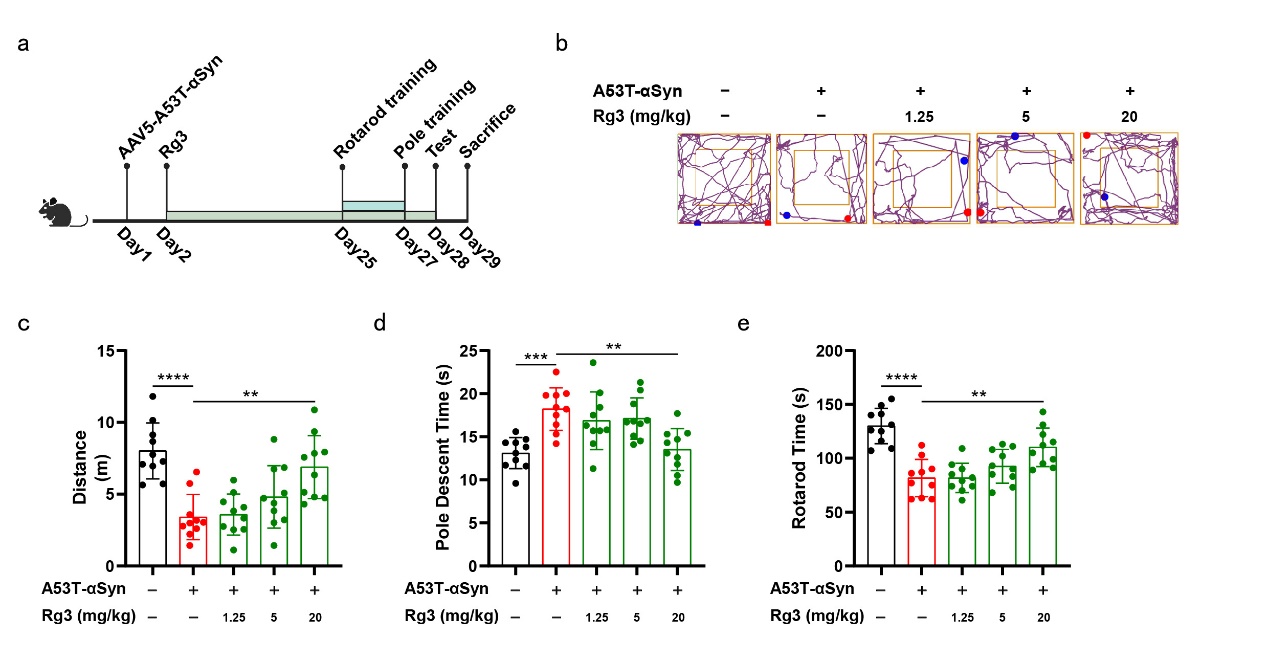


**Supplementary Figure 9**

**(a)** *In vivo* experimental scheme for investigating the therapeutic effects of Rg3. AAV5-*Vector*-injected mice and AAV5-*A53T-αSyn*-injected mice received continuous oral administration of Rg3 (1.25, 5 or 20 mg kg^−1^, i.g.) for 27 days. **(b)** Movement track in the open field test. **(c)** Total distance travelled in the open field test. **(d)** Descent time in the pole test. **(e)** Time-to-fall in the rotarod test (n = 10 mice per group in b–e). Mean ± standard error of the mean is presented. **P < 0.01, ***P < 0.001, ****P < 0.0001. One-way ANOVA with Tukey’s multiple comparisons test (**c**, **d** and **e**). Source data are provided in the Source Data file.


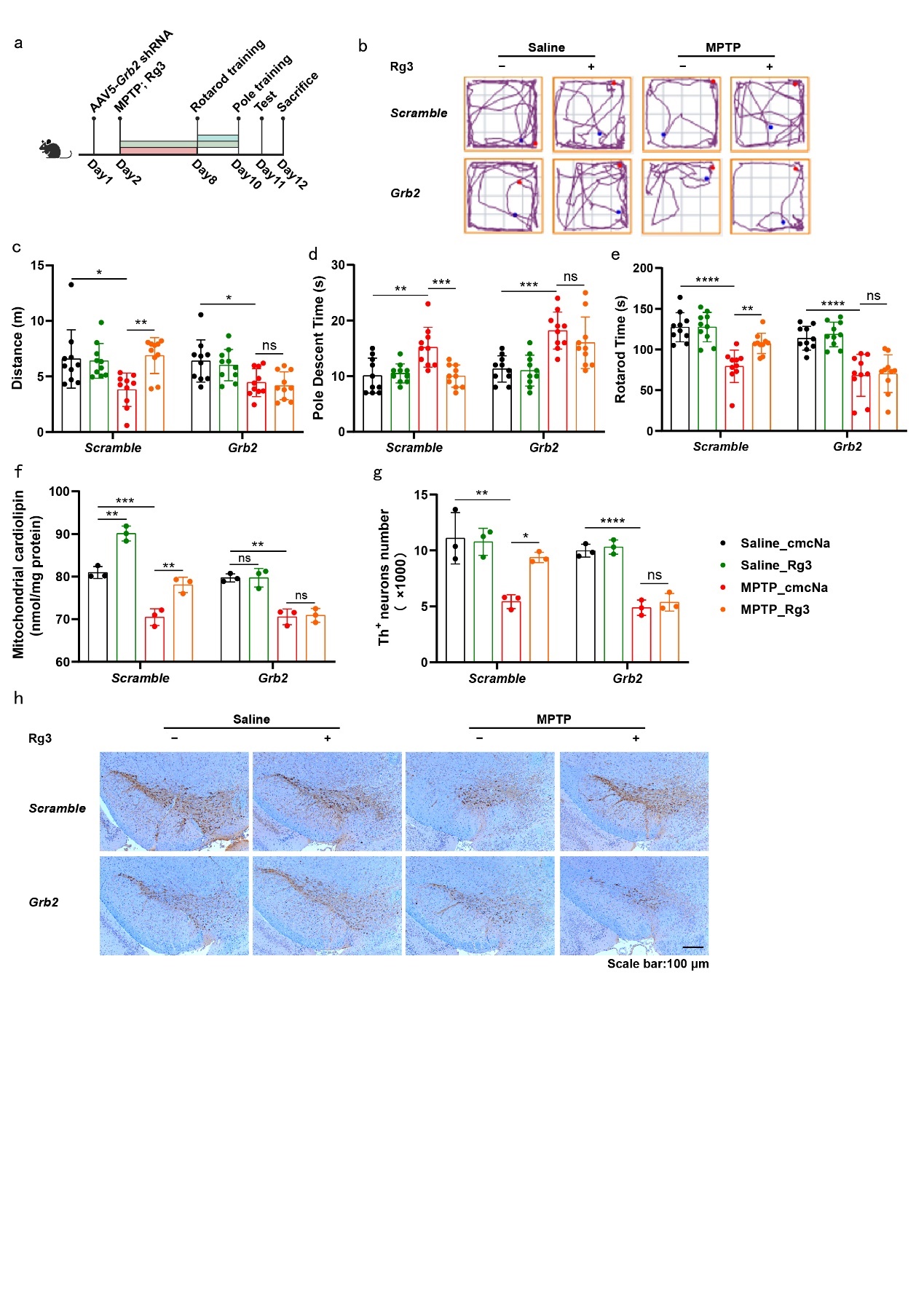


**Supplementary Figure 10**

**(a)** *In vivo* experimental scheme for investigating the therapeutic effects of Rg3. The brain and SN of mice were dissected for further analysis. **(b)** Movement track in the open field test. **(c)** Total distance travelled in the open field test. **(d)** Descent time in the pole test. **(e)** Time-to-fall in the rotarod test (n = 10 mice per group in b–e). **(f)** Mitochondrial CL levels in the SN of mice. **(g)** Quantification of TH^+^ neurons. **(h)** Immunohistochemical staining of TH^+^ neurons (scale bar = 100 µm) (n = 3 mice per group in f–h). Mean ± standard error of the mean is presented. *P < 0.05, **P < 0.01, ***P < 0.001, ****P < 0.0001, ns, not significant. Two-way ANOVA with Sidak’s multiple comparisons test (**c**–**g**). Source data are provided in the Source Data file.


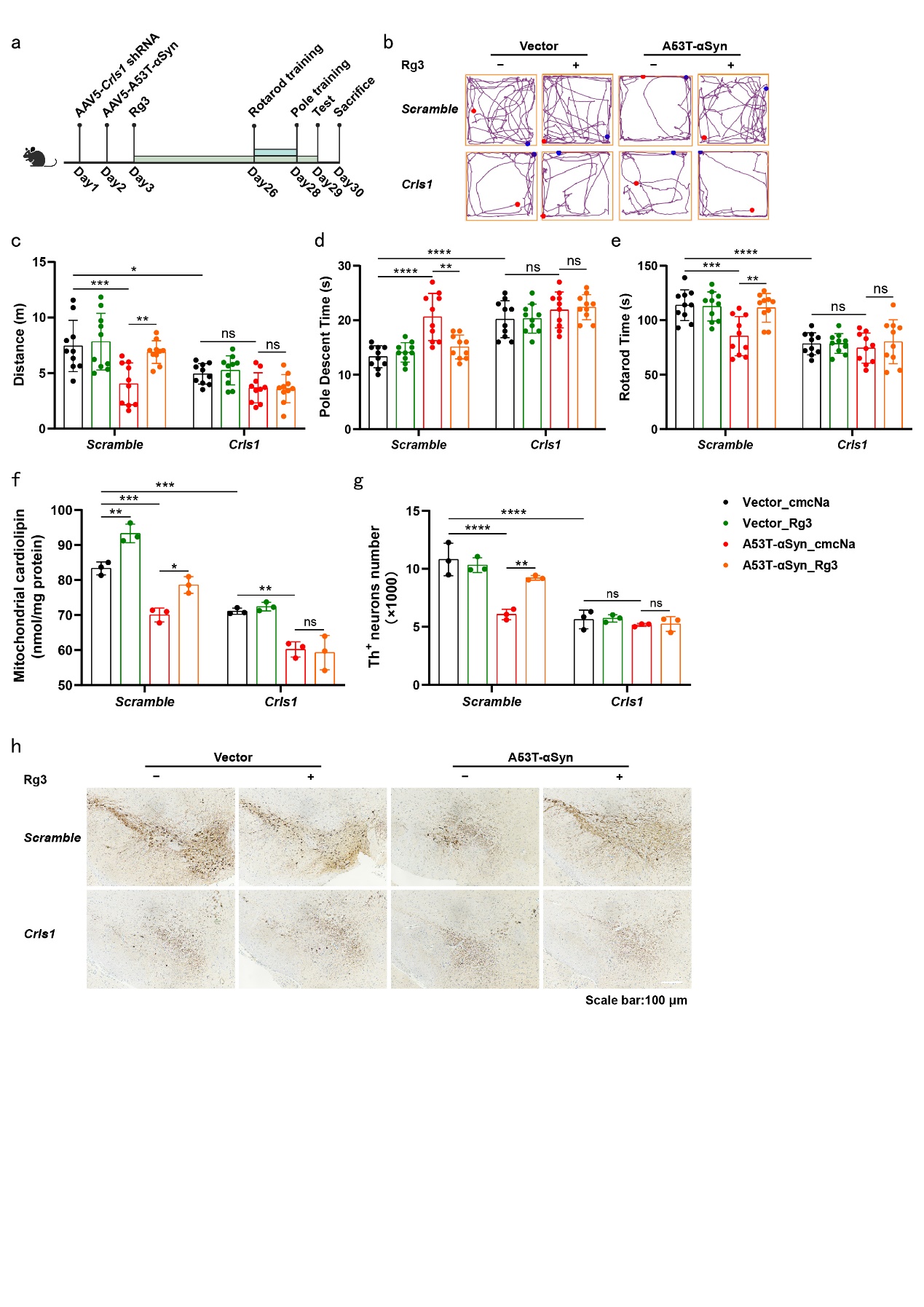


**Supplementary Figure 11**

**(a)** *In vivo* experimental scheme for investigating the therapeutic effects of Rg3. The brain and SN of mice were dissected for further analysis. **(b)** Movement track in the open field test. **(c)** Total distance travelled in the open field test. **(d)** Descent time in the pole test. **(e)** Time-to-fall in the rotarod test (n = 10 mice per group in b–e). **(f)** Mitochondrial CL levels in the SN of mice. **(g)** Quantification of TH^+^ neurons. **(h)** Immunohistochemical staining of TH^+^ neurons (scale bar = 100 µm) (n = 3 mice per group in f–h). Mean ± standard error of the mean is presented. *P < 0.05, **P < 0.01, ***P < 0.001, ****P < 0.0001, ns, not significant. Two-way ANOVA with Sidak’s multiple comparisons test (**c**–**g**). Source data are provided in the Source Data file.


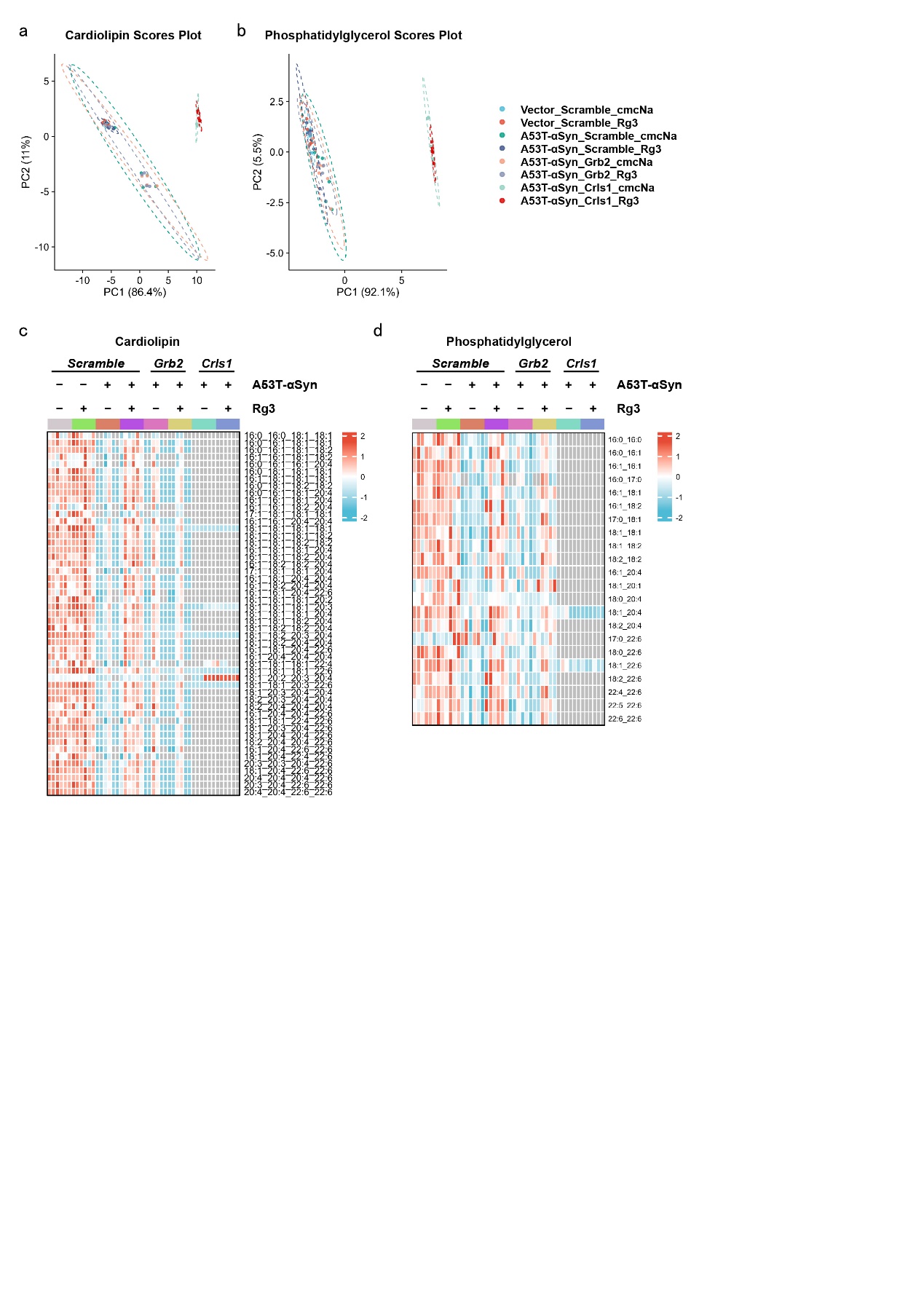


**Supplementary Figure 12**

**(a**, **b)** Principal component analysis scores plot of TIC-normalized log10-transformed intensities for different groups. **(c**, **d)** Heatmap based on peak area parameters derived from MS, mapping various types of CL and PG identified in the metabolite library through non-targeted analysis. n = 6 mice per group. Source data are provided in the Supplementary Table 12 and 13.


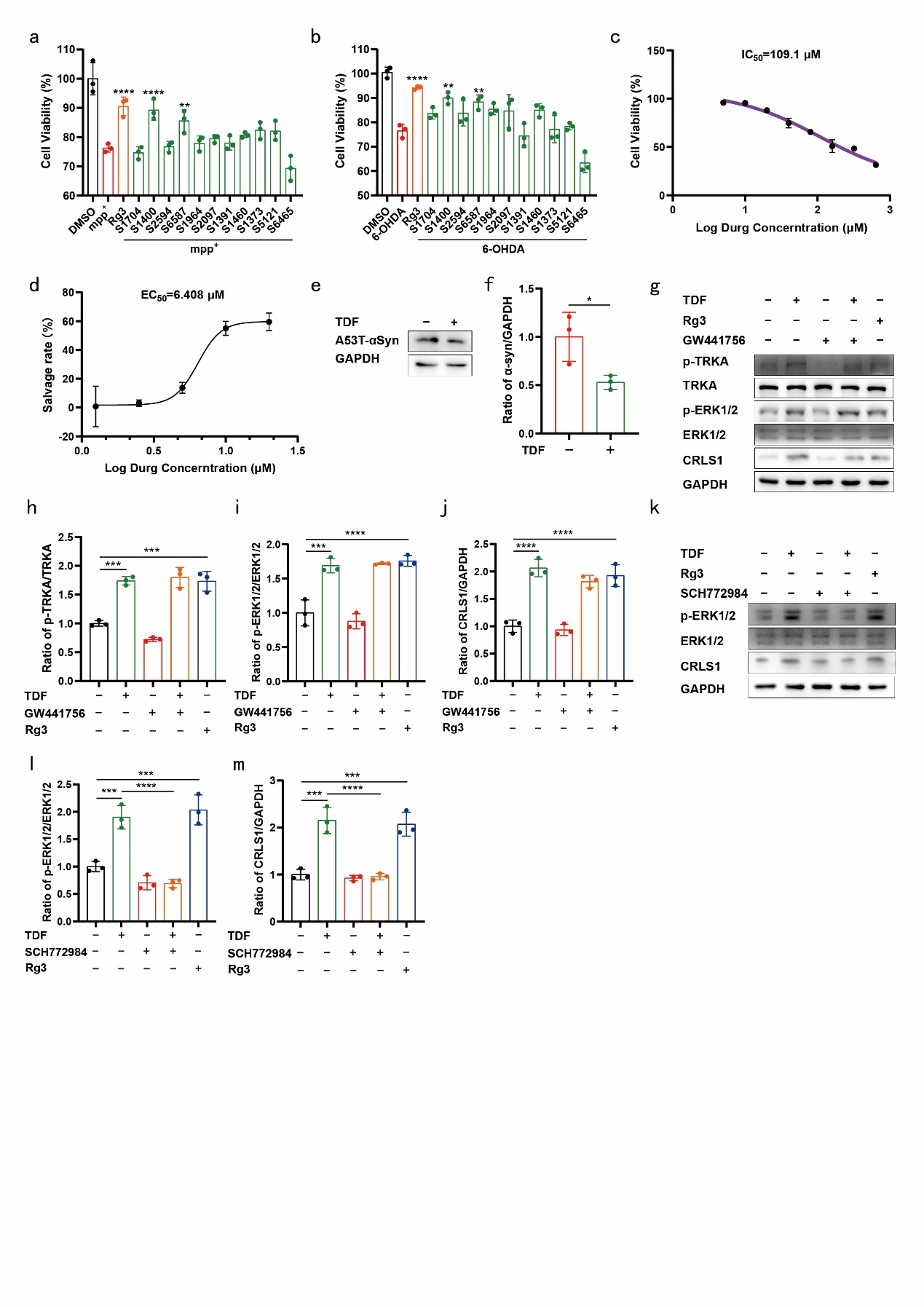


**Supplementary Figure 13**

**(a**–**d)** Cytotoxicity was evaluated via CCK-8 assay. SH-SY5Y cells were treated with mpp^+^ (600 μM), Rg3 (5 μM), indicated compounds (10 μM) or DMSO (0.1%) for 24 h (**a**). SH-SY5Y cells were treated with 6-OHDA (60 μM), Rg3 (5 μM), indicated compounds (10 μM) or DMSO (0.1%) for 24 h (**b**). SH-SY5Y cells were treated with 5-, 10-, 20-, 40-, 80-, 160-, 320- or 640-μM TDF for 24 h. The IC_50_ value of TDF (**c**). SH-SY5Y cells were treated with mpp^+^ (600 μM), 1.25-, 2.5-, 5-, 10- or 20-μM TDF for 24 h. The EC_50_ value of TDF (**d**). **(e**, **f)** SH-SY5Y cells were treated with TDF (10 μM) or DMSO (0.1%) for 24 h after pre-transfection with pCMV3-*Vector* or pCMV3-*A53T-αSyn*-His for 24 h. Representative images of western blotting of A53T-αSyn and GAPDH in SH-SY5Y cells are shown (**e**). Quantification of A53T-αSyn levels (**f**). **(g–j)** SH-SY5Y cells were treated with TDF (10 μM), GW441756 (10 μM) or DMSO (0.1%) for 24 h. Representative images of western blotting of p-TRKA, TRKA, p-ERK, ERK, CRLS1 and GAPDH in cell lysates (**g**). Quantification of p-TRKA levels (**h**). Quantification of p-ERK levels (**i**). Quantification of CRLS1 levels (**j**). **(k–m)** SH-SY5Y cells were treated with TDF (10 μM), SCH772984 (10 μM) or DMSO (0.1%) for 24 h. Representative images of western blotting of p-ERK, ERK, CRLS1 and GAPDH in cell lysates (**k**). Quantification of p-ERK levels (**l**). Quantification of CRLS1 levels (**m**). Three independent experiments per condition were performed in a-m. Data are normalized to DMSO group (**a**, **b**, **f**, **h**, **i**, **j**, **l** and **m**). Mean ± standard error of the mean is presented. *P < 0.05, **P < 0.01, ***P < 0.001, ****P < 0.0001. One-way ANOVA with Dunnett's multiple comparisons test (**a**, **b**) or Tukey’s multiple comparisons test (**h**, **i**, **j**, **l** and **m**), student’s two-tailed unpaired t-test (**f**). Source data are provided in the Source Data file.

**Supplementary Table 1**

List of 1448 proteins (p < 0.05) quantified by label-free mass spectrometry with the potential to bind Rg3.

**Supplementary Table 2**

List of small interfering RNA.

**Supplementary Table 3**

List of qPCR primers.

**Supplementary Table 4**

The hydrogen bonding interaction between GRB2 and TRKA.

The hydrogen bonding interaction between TRKA and GRB2-Rg3 complex.

**Supplementary Table 5**

List of predicted transcription factors of *CRLS1*.

**Supplementary Table 6**

List of primers for EMSA probes.

**Supplementary Table 7**

List of primers for ChIP assay.

**Supplementary Table 8**

The putative target site of ERK in EVI1 protein sequence.

**Supplementary Table 9**

List of compounds screened by SPR technology that bind to GRB2.

**Supplementary Table 10**

List of gene sequences of human GRB2 and TRKA protein.

**Supplementary Table 11**

List of antibodies.

**Supplementary Table 12**

LC/MS data of CL in mouse SN.

**Supplementary Table 13**

LC/MS data of PG in mouse SN.
